# The *STENOFOLIA* gene from Medicago alters leaf width, flowering time and chlorophyll content in transgenic wheat

**DOI:** 10.1111/pbi.12759

**Published:** 2017-06-30

**Authors:** Meiyan Liu, Lei Lei, Fang Miao, Carol Powers, Xiaoyu Zhang, Jungpeng Deng, Million Tadege, Brett F. Carver, Liuling Yan

**Affiliations:** ^1^ Department of Plant and Soil Sciences Oklahoma State University Stillwater OK USA; ^2^ Department of Biochemistry and Molecular Biology Oklahoma State University Stillwater OK USA; ^3^ Present address: School of Life Science Jiangsu Normal University Xuzhou Jiangsu China; ^4^ Present address: College of Life Science Northwest A&F University Yangling Shaanxi 712100 China

**Keywords:** STF gene, leaf width, flowering time, GAGA‐binding protein, protein‐DNA interaction, transgenic wheat

## Abstract

Molecular genetic analyses revealed that the *WUSCHEL‐related homeobox* (*WOX*) gene superfamily regulates several programs in plant development. Many different mechanisms are reported to underlie these alterations. The *WOX* family member *STENOFOLIA* (*STF*) is involved in leaf expansion in the eudicot *Medicago truncutula*. Here, we report that when this gene was ectopically expressed in a locally adapted hard red winter wheat cultivar (*Triticum aestivum*), the transgenic plants showed not only widened leaves but also accelerated flowering and increased chlorophyll content. These desirable traits were stably inherited in the progeny plants. STF binds to wheat genes that have the (GA)_n_/(CT)_n_ DNA *cis* element, regardless of sequences flanking the DNA repeats, suggesting a mechanism for its pleiotropic effects. However, the amino acids between position 91 and 262 in the STF protein that were found to bind with the (GA)_*n*_ motif have no conserved domain with any other GAGA‐binding proteins in animals or plants. We also found that STF interacted with a variety of proteins in wheat in yeast 2 hybrid assays. We conclude that the eudicot *STF* gene binds to (GA)_*n*_/(CT)_*n*_ DNA elements and can be used to regulate leaf width, flowering time and chlorophyll content in monocot wheat.

## Introduction

Postembryonic plant development occurs as a result of pluripotent stem cell activity in the root and shoot apical meristems, which renews the stem cell population while producing other cells that can differentiate into new organs (Kaufmann *et al*., 2010). The leaves in most vascular plants are the primary organs that capture energy from sunlight for photosynthesis, producing carbohydrates that are eventually used, along with other nutrients, for the development, growth and reproduction of the plant (Lemoine *et al*., 2013). Transcription factors play important roles in a diverse range of developmental processes including leaf development. The plant‐specific *WUSCHEL‐related homeobox* (*WOX*) gene superfamily is among the most important groups of transcription factors with diverse functions in plant development (Haecker *et al*., 2004; Mayer *et al*., 1998; Park *et al*., 2005; Rodríguez‐Mega *et al*., 2015; Sarkar *et al*., 2007).

Fifteen members of the *WOX* family have been systematically characterized in the model plant species *Arabidopsis thaliana*, which along with their orthologues in other plant species are divided into three clades based on their conserved protein motifs (van der Graaff *et al*., 2009; Lin *et al*., 2013). The modern clade, which includes *At*WOX1–7, is found in seed plants and contains proteins with two motifs: the homeodomain (HD) and the WUSCHEL (WUS) box. The second is the intermediate clade found in vascular plants such as lycophytes and includes proteins that have several functional motifs but no WUS box, such as *At*WOX8, *At*WOX9, *At*WOX11 and *At*WOX12. The third is the ancient clade found in vascular and nonvascular plants such as mosses and green algae, and contains proteins that have one homeobox (HB) motif and other uncharacterized motifs, including *At*WOX10, *At*WOX13 and *At*WOX14. *WOX* genes regulate some of the most significant phenotypic changes in plants. *AtWOX1* (Fukushima and Hasebe, 2014; Nakata and Okada, 2012; Yamaguchi *et al*., 2012) and its orthologs *STENOFOLIA* (*STF*) in *Medicago trunculata* and *NsLAM1* in tobacco (*Nicotiana sylvestris*) (Tadege *et al*., 2011), *MAEWEST* in *Petunia* (Vandenbussche *et al*., 2009), and *LATHYROIDES* in pea (*Pisum sativum*) (Zhuang *et al*., 2012) play pivotal roles in leaf blade outgrowth. *AtWOX2* and *AtWOX8/9* influence the formation and differentiation of embryos (Haecker *et al*., 2004). *AtWOX3* and its orthologs *NARROW SHEATH1* (*NS1*) and *NS2* in *Zea mays* (maize) affect the development of the lateral stipules of leaves and the lateral sepals and stamens of flowers (Costanzo *et al*., 2014; Matsumoto and Okada, 2001; Nardmann *et al*., 2004). *AtWOX4* regulates the cambium activity in the main stem (Suer *et al*., 2011), and *AtWOX5* influences the root apical meristem activity (Sarkar *et al*., 2007). These associations were determined based on mutant analyses of phenotypic alterations, but the mechanism with which multiple phenotypes in plants are regulated by the *WOX* genes remains unclear.

The *STF* gene in *Medicago truncatula* is expressed in specific leaf cells located in the intersection of abaxial (lower) and adaxial (upper) surface of the leaf blade, thereby mediating leaf blade outgrowth (Tadege *et al*., 2011). At the protein level, STF consists of four major parts: the N‐terminal domain, the HD, the middle domain (MD) and a C‐terminal domain that consists of two highly conserved motifs, a WUS box and the STF box (Zhang *et al*., 2014). The WUS and STF boxes of STF have been reported to physically interact with TPL (TOPLESS), which is a key transcriptional corepressor required for the apical‐basal patterning of the embryo and for shoot development (Long *et al*., 2006; Szemenyei *et al*., 2008; Wang *et al*., 2010). The STF‐TPL complex directly targets the promoter of *ASYMMETRIC LEAVES2* (*AS2*) to promote leaf blade development (Zhang *et al*., 2014). *AS1* and *AS2* have been suggested to be involved in the establishment of adaxial polarity pattering, the formation of the venation system, development of laminar symmetry and the differentiation of leaf cells by repressing the expression of the *KNOTTED1* (*KN1*)‐type homeobox (*KNOX1*) genes that maintain meristem identity in Arabidopsis and maize (Byrne *et al*., 2000; Hake *et al*., 2004; Ori *et al*., 2000; Semiarti *et al*., 2001). The orthologous wheat *TaKN1* genes are known to play important roles in developmental processes by their protein interactions with Bell1‐type homeobox genes (*TaBEL1*) (Mizumoto *et al*., 2011; Takumi *et al*., 2000).

The leaf shape of wild‐type *M. truncatula* is ovate. When *STF* is mutated by the insertion of a transposable element, leaf shape is drastically altered; cell proliferation at the adaxial–abaxial boundary is significantly reduced, leading to a severe decrease in lateral leaf growth and the disappearance of the marginal serrations on the leaf (Tadege *et al*., 2011). Similarly, mutation of *LAM1*, the orthologue of *STF*, in tobacco plants causes leaves to have a narrow, elongated morphology, resembling the narrow strip‐like leaves of hexaploid wheat (Tadege *et al*., 2011). In monocot species, *WOX* orthologs have also evolved functions related to leaf shape; for instance, duplicate genes *NARROW SHEATH1* (*NS1*) and *NS2* regulate the width of the leaf sheath in maize (Nardmann *et al*., 2004), and *NARROW LEAF2* (*NAL2*) and *NAL3* control leaf width in rice (*Oryza sativa*) (Cho *et al*., 2013). Given that the loss of *STF* can change wild‐type ovate Medicago leaves so that they resemble narrow cereal‐like leaves, the above findings raise the intriguing question of whether *STF* can be expressed in wheat to produce wider leaves in this monocot. Wheat cultivars in the southern Great Plains are typically used as dual‐purpose wheat, which requires leaf tissue to be produced for cattle grazing before stem elongation and grain development occurs (Edwards *et al*., 2012; Redmon *et al*., 1996). Larger leaves may produce an improved photosynthetic canopy source in grain‐only wheat, and even more biomass for grazing in dual‐purpose wheat. Even in the absence of grazing, juvenile wheat plants with larger leaves may produce an improved photosynthetic canopy source to drive the transition from vegetative to reproductive tissue formation.

Here, we expressed *M. truncatula STF* in hexaploid wheat and show that transgenic plants display enhanced chlorophyll production, accelerated flowering time, and significantly wider leaf blades compared to controls. We further identified the gene *cis* element in wheat that was directly bound by STF.

## Results

### Transgenic wheat expressing *STF*

**Figure 1:**
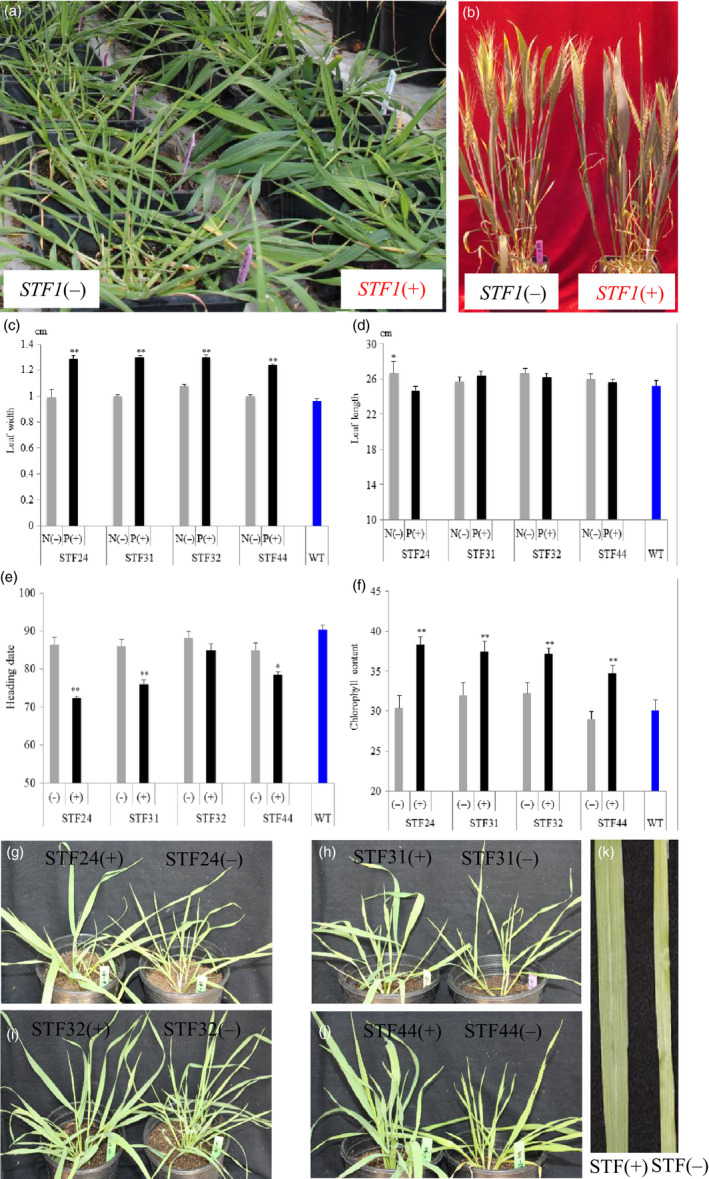
Genetic effects of *STF* in transgenic wheat. (a) Transgenic T_0_ wheat plants expressing *STF*. Plants on the right were positive transgenics, while plants on the left were nontransgenic. (b) Images of T_0_ plants representing a negative control *STF* (‐) on the left, and the transgenic plant STF24 on the right. (c–f) Comparison of phenotypes among the transgenic, nontransgenic and wild‐type (WT, cultivar 2174) plants in four T_1_ families (STF24, STF31, STF32 and STF44) at the juvenile stage. Means ± SD for *n *=* *6‐20 plants derived from each of the four transgenic events. *P <* *0.05 and **P <* *0.001, Student's *t*‐test. The three largest leaves of each plant were measured for (c) Leaf width, (d) Leaf length, (e) Heading date, (f) Chlorophyll content. (g–j) Images of representative T_1_ plants derived from four families, with a positive transgenic plant (left) and its nontransgenic sibling (right), (g) STF24 T_1_ plants, (h) STF31 T_1_ plants. (i) STF32 T_1_ plants, (j) STF44 T_1_ plants. (k) Images of a typical transgenic leaf (left) and its nontransgenic sibling (right).

*STF* orthologues are not found in the sequenced genomes of monocot species including rice, *Brachypodium*, sorghum (Tadege *et al*., 2011) and the recently released genomic sequences of wheat (Data S1). *STF* was transformed into wheat in an attempt to increase leaf size and promote photosynthetic activity. The *ubiquitin* promoter from maize was fused with *STF* in the expression vector pMDC32, and the construct was transformed into the hard red winter wheat cultivar ‘2174’ (PI 602595) using gene gun. Of the 63 resulting plants, 22 were confirmed by PCR to have been transformed with *STF*. Most of the positive T_0_ transgenic plants had wider leaves compared to the nontransgenic plants transformed with the vector alone at similar developmental stages (Figure 1a; Figure S1). Four transgenic T_0_ plants (STF24, STF31, STF32 and STF44) that had the widest leaves at the juvenile stage were self‐pollinated to generate their respective T_1_ transgenic progenies for further studies. All of the T_0_ transgenic plants produced seeds (Figure 1b).

Families of T_1_ progeny plants derived from each of the four T_0_ plants were grown under long days and at a constant temperature in a greenhouse. Twenty‐six T_1_ progeny plants for each of the four families (STF24, STF31, STF32 and STF44) showed an approximately 3 : 1 ratio of transgenic plants carrying the *STF* gene and nontransgenic plants without this gene, indicating the genetic segregation of a single *STF* gene in each of these four families (*X* ^2 ^= 0.05–2.51, *P *< 0.05) (Table S1). The leaf width phenotype clearly segregated in each of these four T_1_ segregating families, with the transgenic plants having significantly wider leaves than nontransgenic siblings and the wild‐type plants at the juvenile stage (Figure S2, Table S2).

In the four T_1_ segregating families, juvenile leaf width increased 20.4%–30.3% among transgenic plants compared with nontransgenic plants derived from the same transgenic event (Figure 1c). Overall, the average leaf width of the transgenic *STF* plants (1.29 cm) increased 25.5%, compared with the nontransgenic plants (1.02 cm). Widened leaves were detectable throughout the entire life cycle of the transgenic wheat plants.

In the adult plant stages, leaf width of the top three leaves of transgenic plants was increased by an average of 20.4%, varying from 13.6% to 25.2%, compared with the nontransgenic plants in all of the four T_1_ families (Figure S3a, Table S2). T_2_ families were generated from the transgenic plants of each T_1_ family, and the widened leaf phenotype was found to be heritable in the T_2_ generation. The transgenic wheat leaves, though wider, were still shaped as strips rather than the ovate morphology (Figure 1k).

The juvenile transgenic plants produced similar leaf lengths compared with nontransgenic plants in three of the T_1_ families (STF31, STF32 and STF44); however, the leaf length of the STF24 transgenic plants was significantly shorter than that of the nontransgenic plants (Figure 1d). In the adult plant stage, the lengths of the uppermost three leaves were similar between transgenic and nontransgenic plants in three T_1_ families (STF24, STF31 and STF32), but leaves of the transgenic plants were significantly shorter in the STF44 family (Figure S3b).

Leaves of *STF* transgenic plants appeared to be greener in colour, compared with nontransgenic siblings (Figure 1g–j). At the juvenile stage, chlorophyll content was increased by an average of 21.3% in transgenic plants relative to nontransgenic plants and the wild type (Figure 1f, Table S2). In the adult plants, however, only the STF44 T_1_ transgenic plants had a significantly higher chlorophyll content than nontransgenic plants (Figure S3c).

Another trait visibly influenced by the *STF* transgene was heading date, which was significantly accelerated in transgenic plants compared with nontransgenic plants, following equivalent vernalization. The average heading date of transgenic T_1_ plants was 14.1 days earlier in STF24 (*P *<* *0.001), 10.0 days earlier in STF31 (*P *<* *0.001) and 6.5 days earlier in STF44 (*P *<* *0.01). No significant difference was found between STF32 plants (Figure 1e, Table S2). Transgenic plants in the T_2_ families followed a similar pattern, with heading dates 7–10 days earlier among transgenic siblings than the nontransgenic and wild‐type plants (Figure S4), although no vernalization was conducted for the T_2_ families (Figure S5a‐d).

### Leaf epidermal cells of transgenic wheat

**Figure 2:**
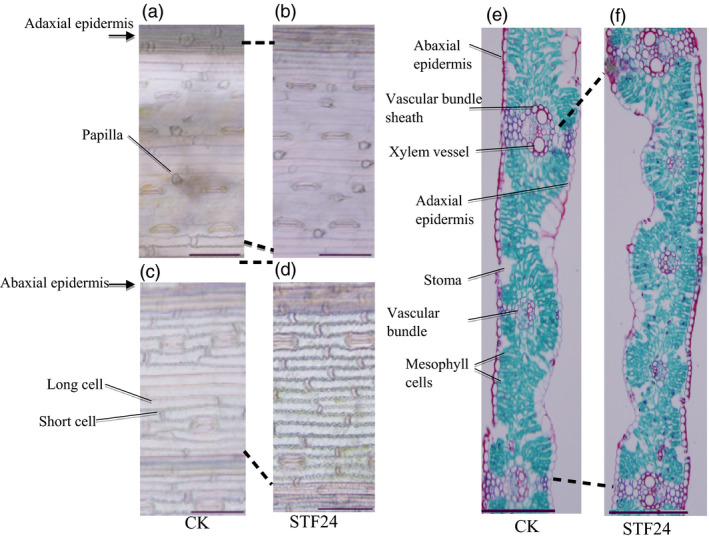
Histological sections of the leaves of transgenic wheat expressing *STF*. (a and b) Adaxial epidermal cells of the flag leaf of wild‐type (a) and transgenic STF24 (b) plants. (c‐d) Abaxial epidermal cells of the flag leaf of wild‐type (c) and transgenic STF24 (d) plants. (e‐f) Transverse section of the flag leaf of wild‐type (e) and transgenic STF24 (f) plants. Images were viewed through a light microscope, with scale bars of 100 μm in (a‐d) and 200 μm in (e) and (f).

To explore the structural mechanism by which *STF* increased wheat leaf width, flag leaf epidermal cells of transgenic versus wild‐type plants were compared. More epidermal cells were observed between two neighbouring veins on the adaxial surface of flag leaves in the transgenic plants (Figure 2a,b). In the interval between the flag leaf middle vein and the neighbouring smaller vein, the number of cells in the transgenic plants is approximately double to that of the wild‐type plants, demonstrating that increased transgenic leaf width was associated with increased cell division. More epidermal cells were also present between veins on the abaxial surface of transgenic plant flag leaves (Figure 2c,d). Transverse sections revealed that wild‐type plants (Figure 2e) produced fewer veins in their flag leaves compared with transgenic plants (Figure 2f), confirming that the wider leaf was caused by an overall increase in cell division in transgenic plants.

### *STF* binds to (GA)_*n*_/(CT)_*n*_ repeats

Three major traits central to wheat growth and development were altered in *STF* transgenic plants; therefore, it was reasonable to hypothesize that transcript levels of multiple genes were directly or indirectly altered by *STF* expression in wheat. The transcriptome profiles of flag leaves from transgenic and wild‐type plants revealed 198 differentially expressed genes (DEGs) between the two genotypes (*P *<* *0.01) (Figure S6). We tested potential DEGs by PCR for their transcripts (Table S3). However, we found a common *cis* site in many DEGs, and we focused on further testing those DEGs that had common *cis* site using quantitative PCR approach.

**Figure 3:**
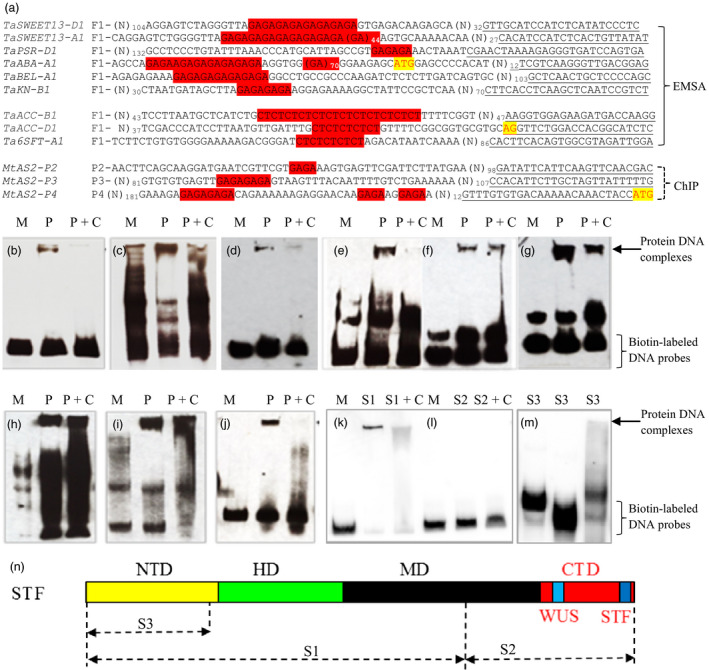
Electrophoretic mobility shift assay (EMSA) interactions of STF protein with the (GA)_n_ DNA probes. (a) Multiple alignments of the sequences used for the biotin‐labelled DNA probe. F1 on the left indicates forward primer sequence (Table S7), and the underlined sequence on the right indicates the reverse primer. The complete sequences of the labelled probes are provided in Figure S7. Nucleotides ATG for the start codon and AG at the 3′ end of an intron are highlighted in yellow and red. Conserved (GA)/(CT) repeats are highlighted in red. Numbers are for repeats of (GA)/(CT). Three promoter fragments (P2, P3, and P4) of *M. trunculata AS2* pulled down using the STF protein and ChIP assays, have (GA)_n_ highlighted in red. (*B‐M*) Images of EMSA interactions of MBP‐tagged STF protein with the (GA)_n_ DNA probes. M indicates a protein VRNA1 (a.a. 85‐end in this study) with an MBP‐tag. *P* indicates the STF protein that was added to EMSA reactions. P+C displays the STF protein and 100X protein competitors that were added to the EMSA reactions. S1, S2 and S3 indicate different segments of the STF protein. (b‐l) DNA probes used in EMSA. (b) *TaSWEET13‐D1,* (c) *TaSWEET13‐A1,* (d) *TaPSR‐D1,* (e) *TaACC‐B1,* (f) *TaACC‐D1,* (g) *Ta6SFT‐A1,* (h) *TaABA‐A1,* (i) *TaBEL‐A1,* (j) *TaKN1,* (k) *Ta6SFT‐A1,* (l) *Ta6SFT‐A1*. (m) The interaction between the S3 STF fragment and, from left to right, *Ta6SFT‐A1*,* TaACC‐D1* and *TaABA‐A1*. (n) A diagram of the three STF segments used in EMSA; S1: a.a. 1‐262, S2: a.a. 263 to the end at a.a. 358, S3: a.a. 1‐90. NTD: N‐terminal domain, HD: homeodomain, MD: middle domain and CTD: C‐terminal domain.

Thirty‐eight genes were found to have the dinucleotide repeat (GA)_*n*_ or its antisense (CT)_*n*_ (where *n* ≥ 4; collectively referred to as (GA)_*n*_ hereafter) within the exon, intron or untranslated region of sequences exported from the International Wheat Genome Sequencing Consortium (IWGSC) databases (Table S4). As a hexaploid, bread wheat usually has three homeologous copies of a gene from its A, B and D genomes. Where one homeologous gene was identified as a candidate DEG, the other two homeologous genes were also analysed. The (GA)_*n*_‐containing fragments were amplified using primers specific to a homeologous gene and confirmed by direct sequencing of the PCR products (Figure 3a). *STF* was expressed in *Escherichia coli*, and the purified STF protein was tested for its interaction with the (GA)_*n*_ element in these genes using an *in vitro* electrophoretic mobility shift assay (EMSA) analysis.

All of the biotin‐labelled (GA)_*n*_ DNA probes analysed (Figure S7) showed direct interactions with the STF protein. *TaSWEET13‐A1* and *TaSWEET13‐D1* are two homeologous genes encoding the sugar transporter SWEET13. *TaSWEET13‐D1* contained a (GA)_8_ motif 82‐100 bp upstream of the start codon, which showed an interaction with STF (Figure 3b), while *TaSWEET13‐A1*, which contained 53 GA repeats, showed a stronger interaction with STF (Figure 3c). *TaPSR‐D1*, encoding photosystem II subunit R, was also shown to interact with STF, even though this probe had only three GA repeats in the promoter, 58‐63 bp upstream of the start codon (Figure 3d).

Probes were also developed for the (GA)_*n*_ DNA elements present in the first intron of two homeologous genes encoding 1‐aminocyclopropane‐1‐carboxylate oxidases (ACC); *TaACC‐B1* contained a (CT)_12_ motif (Figure 3e) and *TaACC‐D1* contained a (CT)_5_ motif (Figure 3f). Another (CT)_5_ motif was found in the second intron of *Ta6SFT‐A1*, which encodes a sucrose:fructan‐6‐fructosyltransferase (Figure 3g). All of the probes developed using intron DNAs having the (GA)_*n*_ elements showed interaction signals with the STF protein.

The (GA)_*n*_ DNA element is also present in numerous cDNAs deposited in GenBank, and its presence in the 5′ untranslated region (UTR) or coding region could imply a role in regulating gene expression. Three homeologous genes for *TaABA1* (*ABA DEFICIENT 1*) were found to have GA repeats in their 5′ UTRs; (GA)_70_ and (GA)_6_ were present in *TaABA‐A1*, (GA)_34_ in *TaABA‐B1*, and (GA)_7_, (GA)_4_ and (GA)_23_ in *TaABA‐D1*. The (GA)_*n*_ DNA element in *TaABA‐A1* was selected to confirm its interaction with STF (Figure 3h).

Genes involved in leaf development were also assessed for the presence of the (GA)_*n*_ DNA element within their promoter sequences and 2 kb of 5′ UTR, as well as their introns. This analysis resulted in the identification of a (GA)_7_ motif present 1210–1223 bp upstream of the start codon of *TaBEL‐A1*, a (GA)_9_ motif upstream of *TaBEL‐B1*, and two motifs (GA)_7_ and (GA)_20_ upstream of *TaBEL‐D1*. STF was confirmed to directly bind to the promoter of *TaBEL‐A1* (Figure 3i). Another (GA)_4_ motif was found in the promoters of the three *TaKN1* homeologs, 120–200 bp upstream of the start codon, and conserved primers were used to amplify *TaKN‐A1*,* TaKN‐B1* and *TaKN‐D1* (together denoted *TaKN1*). STF was confirmed to directly bind to the promoter of these *TaKN1* genes (Figure 3j).

Multiple alignments showed that, other than the (GA)_*n*_ element, the sequences of these STF‐interacting genes were not similar (Figure 3a). This finding suggested that the binding of STF to the DNA fragments occurred at the (GA)_*n*_ element, which was necessary for STF binding, regardless of the sequences flanking the (GA)_*n*_ repeats.

To determine which domain in the STF protein interacted with the (GA)_*n*_ DNA element, three fragments of STF were generated. These fragments included (i) the S1 fragment from amino acid (a.a.) 1 to a.a. 262, (ii) the S2 fragment from a.a. 263 to the end of the protein at a.a. 358 and (iii) the S3 fragment from a.a. 1 to a.a. 90 (Figure 3n). Comparative *in vitro* interactions of the three STF fragments with the same probe showed that the (GA)_*n*_ DNA element interacted with S1 (Figure 3k) but not with S2 (Figure 3l) or S3 (Figure 3m), indicating that the interaction site of STF protein was located between positions a.a. 91 and a.a. 262, which encompasses the homeodomain. However, no specific conserved motif was found between the 91–262 a.a. of STF and other known GAGA‐binding proteins including *At*BPC1‐7 in *Arabidopsis thaliana*,* Gm*GBP1 in Glycine max, and Psq and Trithorax in *Drosophila melanogaster*.

### Regulation of transcriptome‐identified genes containing (GA)_*n*_

**Figure 4:**
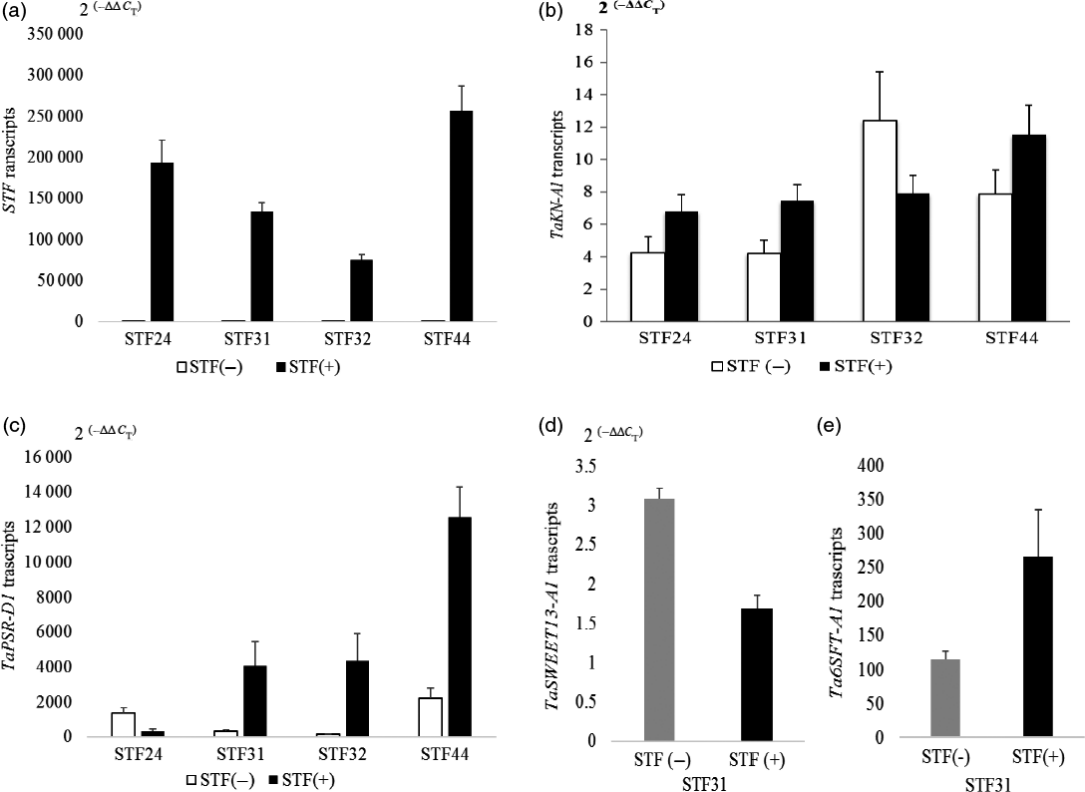
Quantification of gene expression in *STF* transgenic wheat. Transcript levels were calculated by the 2^(−ΔΔ^ ^CT^ ^)^ method, where CT is the threshold cycle. The values represent mean expression levels for *n *=* *15 transgenic plants, and *n *=* *5 nontransgenic plants. Error bars = standard errors. (a) *STF*; (b) *TaKN‐A1;* (c) *TaPSR‐D1;* (d) *TaSWEET13‐A1;* (e) *Ta6SFT‐A1*.

Based on the above results, we considered any gene containing the (GA)_*n*_ DNA element to be a potential target for down‐ or up‐regulation by STF. Accordingly, we analysed the expression levels of (GA)_*n*_‐containing genes in T_2_ transgenic wheat plants expressing *STF* and in nontransgenic controls from the four independent transgenic events, using quantitative PCR. These T_2_ plants were not vernalized to avoid the potential interactive effects of STF with vernalization. As shown in Figure 4a, the transcript levels of *STF* were significantly different among four transgenic events, with a 3.4‐fold difference between plants with the highest transcript levels (STF44) and those with the lowest (STF32).

The transcript levels of several (GA)_*n*_‐containing genes were investigated in the STF T_2_ transgenic plants, to determine the regulatory effect of STF on their expression levels. *TaKN1* transcripts were up‐regulated in transgenic plants relative to the nontransgenic siblings derived from three of the transgenic events (STF24, STF31 and STF44), but not for STF32 (Figure 4b). *TaPSR1* transcripts were up‐regulated in all of the transgenic lines except STF24 (Figure 4c). *TaSWEET13‐A1* transcripts were measured only in the STF31 family, where this gene was down‐regulated by STF (Figure 4d). *Ta6SFT‐A1* transcripts were measured only in the STF31 family, where this gene was up‐regulated by STF (Figure 4e). These results indicated that different genes were down‐ or up‐regulated by STF in the families derived from four different transgenic events.

### Physical interactions of STF with multiple proteins in wheat

It was hypothesized that STF may form protein complexes with additional transcription factors or proteins to promote or repress the expression of genes controlling the three visible traits in wheat. The same cultivar (2174) used for *STF* transformation was used to construct an efficient yeast two‐hybrid (Y2H) library using young shoot tissues including leaves and the shoot apices of vernalized and unvernalized plants. The full‐length STF protein was used as a probe to screen the Y2H library to test for its interactions in wheat.

The resulting 96 positive clones obtained from the screening of approximately 2 × 10^7^ cells were sequenced, with 80 clones producing sequences long enough for further analysis. The sequences of the wheat clones were searched in the NCBI GenBank nonredundant protein database to identify putative proteins, and their corresponding genes were identified in IWGSC databases to determine their chromosomal locations. The closest homologs identified by the BLASTP and IWGSC searches are summarized in Table S5.

Five proteins that repeatedly appeared in the Y2H clones were confirmed to bind directly with STF in plant cells using a transient expression system in tobacco leaves. The five proteins were *Ta*CCDP1 (carbon catabolite‐depressing protein kinase), *Ta*SRP1 (stress responsive protein), *Ta*SUB1 (strubbelig‐receptor family 6), *Ta*6SFT1 and *Ta*STK1, a serine/threonine kinase involved in the control of stomatal movement in response to CO_2_. As the full‐length *STF* was too large to be expressed using the pEG101‐YFP vector or a series of bimolecular fluorescence complementation (BiFC) vectors, STF was split into two protein fragments, STFa (a.a. 1 to a.a. 242) and STFb (a.a. 160 to the final residue, a.a. 358). The five proteins from the Y2H clones, along with STFa and STFb, were investigated in living cells. When *STFa* (Figure S8a) or *STFb* (Figure S8b) alone was expressed, the yellow fluorescent signal was detected predominantly in the nucleus. *Ta*STK1 was detected in both nucleus and cytoplasmic organelles (Figure S8c), while the other four proteins were detected predominantly in the nucleus (Figure S8d‐g).

**Figure 5:**
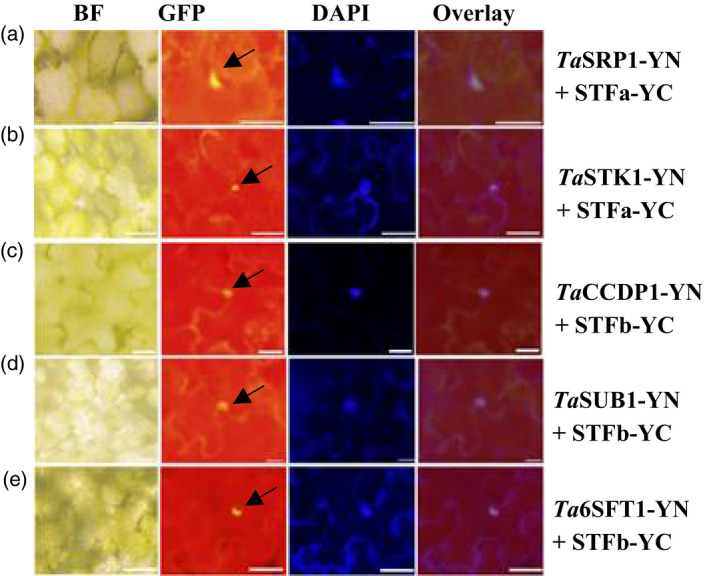
*In vivo* interactions of the STF protein with wheat proteins. *STFa* and *STFb* in a pEG202‐YC vector were transformed with various wheat genes in a pEG201‐YN vector into the leaves of 5‐week‐old *N. benthamiana* plants. Images of leaf discs were taken 3 days after infiltration. (a) *Ta* SRP1‐YN and STFa‐YC; (b) *Ta* STK1‐YN and STFa‐YC; (c) *Ta* CCDP1‐YN and STFb‐YC; (d) *Ta* SUB1‐YN and STFb‐YC; (e) *Ta*6SFT1‐YN and STFb‐YC. Images were taken using a bright filter (BF), a GFP filter, and with an ultraviolet filter (to visualize the DAPI stain of the nucleus). The overlay images align the locations of YFP with the DAPI‐stained nucleus. The scale bar in all images represents 50 μm.

STFa and STFb were independently expressed using the pEG202‐YC vector, while the Y2H proteins (*Ta*STK1, *Ta*CCDP1, *Ta*SRP1, *Ta*SUB1 and *Ta*6SFT1) were expressed using the pEG201‐YN vector. Each of the Y2H‐identified proteins was independently tested for their interaction with STFa or STFb by performing BiFC assays. *Ta*SRP1‐YN (Figure 5a) and *Ta*STK1‐YN (Figure 5b) showed strong interactions with STFa‐YC, while *Ta*CCDP1‐YN (Figure 5c), *Ta*SUB1‐YN (Figure 5d) and *Ta*6SFT1‐YN (Figure 5e) showed strong interactions with STFb‐YC. These results indicated that the STF protein has one domain between a.a. 1 and a.a. 160 that interacted with *Ta*SRP1 and *Ta*STK1, and another domain between a.a. 242 and a.a. 358 that interacted with *Ta*CCDP1, *Ta*SUB1 and *Ta*6SFT1 proteins in wheat.

## Discussion

In this work, we found that when the *STF* gene from the eudicot *M. truncatula* was ectopically expressed in bread wheat, a monocot species, the most striking difference observed between the transgenic and nontransgenic siblings was widened leaves, particularly at the juvenile stage. This finding indicates that the key role of *STF* in the determination of leaf width was maintained when expressed in an unrelated monocot species. In addition, two other traits, flowering time and leaf greenness, were modified in the transgenic wheat plants. Phenotypic variation in these traits among the four transgenic events could be caused by differential transcript levels of *STF*. Variation in *STF* transcript levels was probably due to the location of insertion site for a given transgenic event. The (GA)_*n*_ DNA motif present in numerous genes in wheat was directly bound by the STF protein expressed in the various transgenic events. The number of GA repeats in a gene is important for its interaction with STF; the more GA repeats a gene had, the more affinity the STF proteins had to these binding sites, affecting the magnitude of its level of activation or repression for a (GA)_*n*_‐containing gene and thus growth and development traits that are regulated by this gene.

STF directly bound to (GA)_*n*_ DNA elements in the promoter, introns or 5′ UTRs of the target genes tested in the EMSA assays in this study. Three independent DNA fragments from different regions of the proximal promoter (P2, P3 and P4) of *AS2* in *M. truncatula* were pulled down by the STF protein using chromatin immunoprecipitation (ChIP) assays (Zhang *et al*., 2014). Interestingly, these *AS2* promoter sequences contain the (GA)_*n*_ repeats (Figure 3a), corroborating the direct binding of STF to the (GA)_*n*_ DNA element in plants.

It has been reported that *STF* may be involved in modulating signals from auxin, cytokinin and sugar metabolism to affect leaf development (Tadege and Mysore, 2011; Tadege *et al*., 2011). Among the genes known to affect leaf development in wheat, *TaKN1* and *TaBEL1* (Mizumoto *et al*., 2011; Takumi *et al*., 2000), were indeed found to share the common structural feature of (GA)_*n*_ repeats in their proximal promoters or other regulatory regions. *Ta6SFT1* and *TaSWEET13*, both involved in sugar metabolism, contain (GA)_*n*_ elements at their regulatory sites. STF not only bound directly to the regulatory sites of *Ta6SFT1*, but also had a physical interaction with its protein, suggesting that STF may also be involved in the regulation of sugar metabolism in a feedback loop. This may also suggest a potential mechanism by which the chlorophyll content and heading dates were altered in the transgenic *STF* wheat plants; chlorophyll biosynthesis and photosynthetic efficiency are regulated by sucrose in carrot (Edelman and Hanson, 1971), and it is generally accepted that Arabidopsis plants, as well as temperate cereals such as wheat and barley, do not flower until they accrue adequate sugar reserves (Hendry, 1993). These results suggest that variation in these visible morphological traits as well as other physiological traits in the transgenic plants might be a consequence of multiple genes regulated by *STF*.

A particularly interesting phenomenon observed in this study is that nontransgenic plants were visibly different from the wild‐type plants. These changes were fairly consistent in the four independent transformation events; therefore, it seems unlikely that these changes were caused tissue culture‐mediated soma clonal variation such as activation of transposons and retrotransposons. It is also likely that insertion of the vector DNA may have resulted in systematic changes to miRNAs or caused epigenetic modifications of certain genes. The difference in leaf length between the transgenic and nontransgenic plants derived from STF24 (Figure 3d), for example, could be caused by such changes to a gene related to leaf length in line STF24.

In Medicago, STF interacts with TPL to repress the expression of the AS2, and regulate leaf development, with the WUS box and STF box in the C‐terminal region of STF interacting with TPL (Zhang *et al*., 2014). No orthologous *TPL* gene was found in the clones identified from the wheat Y2H library using STF as a bait. STF may have additional functions when it interacts with the proteins identified from the wheat Y2H library, such as *Ta*SUB1. In Arabidopsis, SUB1 is a temperature‐sensitive receptor‐like kinase that plays a role in coordinating cell proliferation and differentiation during leaf development (Lin *et al*., 2012). The function of *TaSUB1* in wheat is yet to be characterized; it would be interesting to investigate if *Ta*SUB1 phosphorylates STF in wheat.

The first protein known to bind to a GAGA motif is encoded by the gene *Trithorax* in *Drosophila melanogaster* (Soeller *et al*., 1993). In plants, the first GAGA‐binding protein discovered was *Gm*GBP1 (GAGA‐binding protein) in soybean (*Glycine max*), which was identified in a yeast one‐hybrid library screen for proteins that bind to a (GA)_9_ DNA motif in the promoter of *GSA1* (*GLUTAMATE 1‐SEMIALDEHYDE AMINOTRANSFERASE*) (Sangwan and O'Brian, 2002). The Arabidopsis GAGA motif binding factor (GAF) proteins comprise the seven BASIC PENTACYSTEINEs (BPCs) (Meister *et al*., 2004; Santi *et al*., 2003; Simonini and Kater, 2014). In this study, we uncovered that STF, which belongs to the WOX family, binds to (GA)_*n*_ repeats similar to BPCs. Furthermore, the amino acids in STF that bind (GA)_*n*_ appear to be located between position a.a. 91 and a.a. 262. However, sequence comparison showed no conserved domain between STF and other GAGA‐binding proteins in animals or plants that enables them to bind to the GA repeats.

This study provides an example of a genetic master regulator from eudicot species being successfully used to generate a desirable trait in a monocot species. The diversification of morphologies within and among species is one of the oldest biological problems to unravel (Carroll, 2008; Muller, 2007; Stern, 2000; Willmore, 2012). The results of this study indicate that a single transcription factor, STF, can generate diversity for several traits in wheat, suggesting that transcriptomic and proteomic analyses of transcription factors can be used to establish a network of genetic to phenotypic alterations leading to the emergence of novel traits.

## Experimental procedures

### Transgenic wheat generation and characterization

*STF* was previously fused into a pMDC32 vector that has 2 × 35S promoter (Tadege *et al*., 2011). In this study, the 35S promoter was replaced by a maize *ubiquitin* promoter (Christensen *et al*., 1992), which is universally expressed and has previously been successfully used to express genes in wheat (Yan *et al*., 2006). The *ubiquitin* promoter was inserted between the *Hind* III and *Kpn* I sites in pMDC32. The *ubiquitin*‐*STF*‐ pMDC32 construct was transformed into 2174 embryos by micro‐projectile bombardment as described previously (Okubara *et al*., 2002). Transformants were selected using shoot regeneration media and rooting media containing 25 mg/L hygromycin.

The T_1_ seeds from five T_0_ positive plants (STF24, STF31, STF32, and STF44 and STF47) were germinated and grown in a greenhouse at 20–25 °C and in a long‐day photoperiod (16/8 h light/dark), with one plant in each pot (diameter: 10 cm, height: 12 cm) containing 1.8 kg commercial soil (Sun Gro Horticulture Canada Ltd., Agawam, MA). Plants at the 4th leaf stage were moved to a cold room at 4 °C in long days to vernalize for 6 weeks, then moved back to 20–25 °C. The T_1_ plants were grown on 8 April 2015, and their leaf widths and lengths were measured at the juvenile stage (29 May) and at the adult stage (6 July). Chlorophyll content was measured on 9 June and on 6 July using a SPAD 502 Chlorophyll Meter (Konica Minolta Sensing Inc., Osaka, Japan). The juvenile plants were photographed on 9 June. Histological sections of the leaves of transgenic wheat expressing *STF* were analysed using the method as described in Chen *et al*. (2016). Each of three positive T_1_ plants derived from each transgenic event was used to generate one T_2_ family, and nontransgenic and wild‐type plants were used as controls. The T_2_ plants were planted on 30 October 2015 in the long‐day conditions at 20–25 °C without vernalization.

### Transcriptome profiling

The transcriptome profiles of the transgenic plant flag leaves were analysed to identify the genomewide targets of STF using next‐generation RNA sequencing. Sequencing libraries were generated from total RNA using an Illumina TruSeq Stranded mRNA sample prep kit (Illumina, Inc., San Diego, CA) by SeqMatic, LLC (San Francisco, CA). Reads were aligned to *T. aestivum* cDNA sequences using Bowtie2. Reads mapping to cDNA sequences were counted, and an unreplicated differential expression analysis was performed using DESeq, resulting in the identification of 198 cDNAs with a *P*_adj_ value of less than 0.01.

### EMSA

The gDNA fragment used as a probe was amplified using Phusion High‐Fidelity DNA Polymerase (New England BioLabs, Ipswich, MA) using the EMSA probe primers listed in Table S7. A Pierce™ Biotin 3′ End DNA Labeling kit (Thermo Fisher Scientific, Waltham, MA) was used to label the 3′‐OH end of the double‐stranded DNA. Four cDNAs were generated to encode STF protein fragments and were amplified using the primers listed in the Table S6. These cDNAs were cloned into a pMAL‐c2X vector with an MBP‐tag (New England BioLabs), and the constructs were then co‐transformed with a Rosetta vector and expressed in *E. coli* (BL21 DE3). An amylose column (New England BioLabs) was used to dialyse and purify the proteins fused with MBP‐tag. Purified proteins MBP‐STF S1 (a.a. 1‐262), MBP‐STF S2 (a.a. 263‐358) and MBP‐STF S3 (a.a. 1‐90), as well as the full MBP‐STF protein (a.a. 1‐358; S), were used in the EMSA.

The EMSA was performed with the Light Shift^®^ Chemiluminescent EMSA Kit (Thermo Fisher Scientific). The binding reaction was performed using a solution containing 10 mm TRIS (pH 7.5), 50 mm KCl, 1 mm DTT, 2.5% glycerol, 0.05% NP‐40, 5 mm MgCl2, 0.5 mm EDTA, 5 ng/μL poly (dI.dC), 1 μg recombinant fusion protein and 100 fmol biotin‐labelled DNA. The reaction mix was kept at room temperature for 40 min before a loading buffer was added. A total volume of 20 μL of the reaction mix was loaded into 6% native polyacrylamide gel in electrophoresis. After blotting on a positively charged nylon membrane (Amersham BioSciences, Amersham, UK), the DNA was linked using a UV‐light cross‐linker (VWR) equipped with 254 nm bulbs at 120 mJ/cm^2^ for 45 s. The membrane was exposed to a FluorChem System (ProteinSimple, San Jose, CA) for 1.5–5 s.

### Quantitative RT‐PCR

Total RNA was extracted from the leaves using TRIzol^®^ reagent (Invitrogen. Carlsbard, CA). The cDNA was synthesized from 1 μg RNA treated with Deoxyribonuclease I using a SuperScriptTM II Reverse Transcriptase kit X and with an oligo(dT)_20_ primer (Invitrogen). Quantitative RT‐PCR (qRT‐PCR) was carried out on a 7500 Real‐time PCR System (Applied Biosystems, Foster City, CA) using iQTM SYBR^®^ Green Supermix (Bio‐Rad Laboratories, Hercules, CA), with actin used as an endogenous control. The primer sequences for all amplified genes are summarized in Table S7. At least six technical repeats and three biological repeats were performed for each sample.

### Protein–protein interactions

The cDNA encoding the complete STF protein was amplified from a previous construct (Tadege *et al*., 2011) using primers STF‐EcoRI‐F1 and STF‐BamHI‐R1. *STF* was cloned into the DNA‐binding domain of the pGBKT7 vector, and the construct was transformed into the yeast strain Y187. The expressed protein was used as ‘bait’ to screen a Y2H ‘prey’ library constructed from the wheat cultivar 2174. The Y2H library was constructed using the ‘Matchmaker™’ Library Construction & Screening System (Takara Bio USA, Inc., Madison, WI)**,** and RNA was extracted from the pooled samples of young leaves and the shoot apexes of plants vernalized for one, two and 3 weeks (Cao and Yan, 2013). Approximately 2 × 10^7^ cells within the original library were used to screen for proteins that interact with STF after being cultured at 30 °C for 5 days. The positive colonies were screened, further confirmed and selected for sequencing using a previously reported protocol (Li *et al*., 2013). The sequences of the cDNA clones were searched for in the wheat nucleotide sequence NCBI expressed sequence tag (EST) database to characterize the protein interactors.

Primers STF‐EcoRI‐F1 and STF‐BamHI‐R were used to amplify a cDNA encoding STF1a (a.a. 1‐242), while STF‐EcoRI‐F and STF‐BamHI‐R1 were used to amplify the cDNA encoding STF1b (a.a. 160 to the end at a.a. 358), and the primers are listed in Table S6. The cDNA fragments identified from the Y2H library were amplified using the primers listed in Table S6. These cDNAs were cloned into the vector pDONR207 with the BP cloning kit (Invitrogen). An LR cloning kit (Invitrogen) was used to transfer each fragment to the pEarleygate101 vector (pEG101) for the analysis of their subcellular localization.

The localizations of proteins were investigated in living cells. Protein interactions were analysed using BiFC. STFa and STFb in pDONR207 were, respectively, fused to the N‐terminal 174 amino acid portion (a.a. 1–174) of yellow fluorescent protein (YFP) in the pEarleyGate202‐YC vector (pEG202‐YC) to test their *in vivo* interaction with each of the proteins identified from the Y2H screen, which were fused to the C‐terminal amino acid portion (a.a. 175–239) of YFP in the pEarleyGate201‐YN vector (pEG201‐YN). Empty vectors were also used as negative controls for *in vivo* interactions. Leaf discs were imaged using the same protocol as previously reported (Li *et al*., 2013).

### Unique sequences in STF for binding to the (GA)_*n*_ element

The STF protein sequences between a.a. 91 and a.a 262 a.a. characterized as binding to the (GA)_*n*_ element were used to check for any conserved domains that may exist in GAGA‐binding proteins in plant and animals. The GAGA‐binding proteins include *At*BPC1 (AAR28441), *At*BPC2 (ABC25617), *At*BPC3 (AAY34178), *At*BPC4 (AAR25824), *At*BPC5 (OAO96752), *At*BPC6 (NP_568605) and *At*BPC7 (NP_181098) in *Arabidopsis thaliana*,* Gm*GBP1 in Glycine max (GenBank no. AAM27201) and Psq (AAC47153) and Trithorax in Drosophila melanogaster (AAA16072).

## Supporting information


**Figure S1** Transgenic T_0_ wheat plants expressing *STF*.
**Figure S2** Images of representative transgenic T_1_ plants.
**Figure S3** Comparison of phenotypes between the transgenic plants and non‐transgenic plants in four T_1_ populations.
**Figure S4** Comparison of heading date between the transgenic plants, non‐transgenic plants, and the wild type.
**Figure S5** Images of representative transgenic T_2_ plants.
**Figure S6** Heatmap of Top 100 Genes/Transcripts by *P*‐value.
**Figure S7** The complete sequences of the DNA probes used for interactions with STF.
**Figure S8** Subcellular localization of STF and its interacting proteins.
**Table S1** Chi‐squared Goodness of Fit for segregation between the presence of *STF* and the absence of *STF* in four T_1_ populations.
**Table S2** The significance of difference in leaf width, chlorophyll content and heading date between transgenic and non‐transgenic plants.
**Table S3** Primers used to test DEGs.
**Table S4** Genes with (GA)_*n*_/(CT)_*n*_ (*n* ≥ 4) identified using the transcriptome sequences or introns or untranslated regions exported from IWGS databases.
**Table S5** Putative STF interacting proteins identified in a 2174 Y2H library.
**Table S6** Primers for protein‐protein and protein‐DNA interactions.
**Table S7** Primers used for EMSA probes and gene expression.
Data S1. No orthologue of *STF* in genomic sequences of wheat
