# Supplementary material for: The STENOFOLIA gene from Medicago alters leaf width, flowering time and chlorophyll content in transgenic wheat
Source: Plant Biotechnol J. 2017 Jun 30;16(1):186–96. doi: 10.1111/pbi.12759 (PMC5785358; doi:10.1111/pbi.12759)
Supplement: Supplementary file 1 — Figure S1 Transgenic T0 wheat plants expressing STF. Figure S2 Images of representative transgenic T1 plants. Figure S3 Comparison of phenotypes between the transgenic plants and non‐transgenic plants in four T1 populations. Figure S4 Comparison of heading date between the transgenic plants, non‐transgenic plants, and the wild type. Figure S5 Images of representative transgenic T2 plants. Figure S6 Heatmap of Top 100 Genes/Transcripts by P‐value. Figure S7 The complete sequences of the DNA probes used for interactions with STF. Figure S8 Subcellular localization of STF and its interacting proteins. Table S1 Chi‐squared Goodness of Fit for segregation between the presence of STF and the absence of STF in four T1 populations. Table S2 The significance of difference in leaf width, chlorophyll content and heading date between transgenic and non‐transgenic plants. Table S3 Primers used to test DEGs. Table S4 Genes with (GA) n /(CT) n (n ≥ 4) identified using the transcriptome sequences or introns or untranslated regions exported from IWGS databases. Table S5 Putative STF interacting proteins identified in a 2174 Y2H library. Table S6 Primers for protein‐protein and protein‐DNA interactions. Table S7 Primers used for EMSA probes and gene expression. Data S1. No orthologue of STF in genomic sequences of wheat [file PBI-16-186-s001.pdf]

Supplementary Information

No orthologue of *STF* in genomic sequences of wheat

The *STF* DNA sequence was used to search in the genome databases recently released by the International Wheat Genome Sequencing Consortium (IWGSC) or NT/EST databases in GenBank. Even though the threshold was reduced from normally used 0.0001 to 0.1, no similar gene was gained except a few fragments that showed low identity within less than 30 bp range. This result suggested that wheat might have no orthologue of *STF*. When the STF protein sequence was used to search in protein databases in GenBank, it showed 54% identity or 68% similarity to TaWOX5 (KF038329) for root development (Zhao et al., 2014), but deduced wheat TaWOX5 protein was found not to have either WUS or STF box of STF.

Supplementary Tables

Table S1. Chi-squared Goodness of Fit for segregation between the presence of *STF* and the absence of *STF* in four T_1 populations

	STF presence	*STF* absence	Total	X^2
STF24	16	10	26	2.51
STF31	20	6	26	0.05
STF32	16	10	26	2.51
STF44	17	9	26	1.28

T_1 plants were identified by using PCR, and transgenic and non-transgenic plants were analyzed by the chi-squared method.

Table S2. The significance of difference in leaf width, chlorophyll content and heading date between transgenic and non-transgenic plants

STF		Leaf width (cm)		Chlorophyll content (reading value)		Heading date (days)	
**Transgenic events**	**Statistic analysis**	**Transgenic**	**Non-transgenic**	**Transgenic**	**Non-transgenic**	**Transgenic**	**Non-transgenic**
**STF24**	**Average**	**1.294**	**0.988**	**36.32**	**35.44**	**72.3**	**86.4**
	**Standard Deviation**	**0.106**	**0.294**	**3.96**	**2.41**	**1.6**	**8.55**
	**Standard error**	**0.016**	**0.068**	**1.33**	**0.92**	**0.44**	**2**
**STF31**	**Average**	**1.307**	**1.015**	**41.25**	**41.07**	**76**	**86**
	**Standard Deviation**	**0.108**	**0.069**	**5.46**	**2.23**	**4.65**	**6.43**
	**Standard error**	**0.014**	**0.012**	**1.54**	**0.64**	**1.08**	**1.9**
**STF32**	**Average**	**1.3**	**1.081**	**40.29**	**38.53**	**85**	**88.2**
	**Standard Deviation**	**0.116**	**0.064**	**1.51**	**1.82**	**6.99**	**6.42**
	**Standard error**	**0.015**	**0.01**	**0.55**	**0.69**	**0.23**	**1.67**
**STF44**	**Average**	**1.238**	**1**	**38.79**	**32.67**	**78.5**	**85**
	**Standard Deviation**	**0.073**	**0.075**	**3.91**	**6.23**	**3.23**	**6.27**
	**Standard error**	**0.01**	**0.012**	**0.83**	**1.07**	**0.78**	**1.77**

Table S3. Primers used to test DEGs

DEGs	Potential function	Primer name	Primer sequences (5'---3')
Traes_2AS_AE82C22DB.1	EMF2	STF-DN1-F1	AGCCCTTGATAATCCCGCT
		STF-DN1-R1	GTGGTCTTTACTAGCAAATTCAGTG
TRAES3BF021500010CFD_t1	Gibberellin 20 oxidase 2	STF-DN2-F1	AAGGACGTGACGAAGCGA
		STF-DN2-R1	GCCCGTTCGACAGAGCCATGAAG
Traes_4AL_2FF7E3B18.1	Cyclin-like F-box	STF-DN3-F1	GGATCAGCTCGACCGGGAA
		STF-DN3-R1	ACAGATCCGCCTGGAGCC
Traes_6DL_1CCB8CC4B1.1	Photosystem II sub-R	STF-DN4-DF1	GAACGAGCGAAACTCCGAGGTT
		STF-DN4-DR1	GTGTATATATTGGGCTGTACCCATCAACA
Traes_6DL_0A92A2224.1	Photosystem II CP43	STF-DN5-F1	AAAATGACTACAATTTTGGGTACC
		STF-DN5-R1	GGAAATAACCAAATATAACACTAGGG
Traes_4AL_450759DB9.1	S-F-6-fructosyl	STF-DN6-F1	TCGGTGGCGACGACAAC
		STF-DN6-R1	CGTCCTTGGAACTGACTGG
Traes_5BL_91FBE67D3.1	Fructokinase-2	STF-DN7-F1	CTTCGTTGGCAAGTTTGGCGAC
		STF-DN7-R1	AGGAGCATGTCGGCGGAT
Traes_4BL_1F7EA23DE.1	Cytochrome c oxidase	STF-DN8-F1	ATGACAAATATGGTTTGATGT
		STF-DN8-R1	AGTGATGATAGGTGGATGTGGT
Traes_2BL_85629BCE9.1	Polyphenol oxidase	STF-DN9-F1	CTCTGCGCCATGGACCAA
		STF-DN9-R1	GGCTGCTTCAGAGACTACAG
Traes_5DL_1522B03F7.1	Astaxanthin syn	STF-DN10-F1	TTCAAACGGGCACACCACT
		STF-DN10-R1	CTTCCATCGTCAATTTCCATATG
TRAES3BF002700090CFD_t1	TCP Transcription factor	STF-UP1-F1	TTGCGTCCACGCTCGCCC
		STF-UP1-R1	GCGGCGGCAGCTATG
Traes_5AS_33A8004A5.1	Nitrogen fixation NifU	STF-UP2-F1	GCTATCACGGACAAGGAC
		STF-UP2-R1	TTTTGCAGTTGAAGTTTACGGCAG
Traes_6AL_EAF53EB94.1	SWEET13	STF-UP3-F1	GACAAATACGTCGCGATGGC
		STF-UP3-R1	GCCGGAACGGCGGTGACT
Traes_4AL_D0DECE300.2	Gibberellin 20 oxidase 1	STF-UP4-F1	GACCTCCCGCGATGCAGAG
		STF-UP4-R1	GCTCGGGCAGGGCGGA
Traes_6BL_415F13B1D.1	Disease resistace RPM1	STF-UP5-F1	GTTAGTGACAAAGATGAAGAGCCAA
		STF-UP5-R1	TCTAGAGTCAACGGTCGCATTT
Traes_4AL_148DC1816.1	Chaperone protein	STF-UP6-F1	TGTCTTAAATGTGACTCAGGC
		STF-UP6-R1	GGACATATTGGTCTCCATAAGAATAT
Traes_6DL_20A343A96.2	SPX membrane	STF-UP7-F1	GGCTATCGGTTTACAGATTATTACG
		STF-UP7-R1	CCTTCAAGGTGATTGATGGG
TRAES3BF048200070CFD_t1	DEAD-box ATP	STF-UP8-F1	CAGATCTTGTGAAGATTTTGGAGC
		STF-UP8-R1	GTCACCACCACGGTTAGA
Traes_1AL_CEA78C84D.2	Pollenless3	STF-UP9-F1	ATCACCAAGACGGCCCGC
		STF-UP9-R1	TCGTCATCCACATCCTCGAGT
Traes_4DL_92CE4A8D1.1	Thioredoxin	STF-UP10-F1	CATCTGCCCGTTTGCTCAACGT
		STF-UP10-R1	AAGCTCTCTCCGGTGACCC

Table S4. Genes with $(GA)_n/(CT)_n$ ($n \geq 4$) identified using the transcriptome sequences or introns or untranslated regions exported from IWGS databases

	Total	Exon	Intron	Outside of ATG
$(GA)_4$	11	Traes_1BS_CEBFF8BEB Exon 1	Traes_2DS_032B6DE74.4 Intron 1	Traes3BF021500010CFD_t1
		Traes_1DS_92D0315C6.2 Exon 4	Traes_6BL_D85A25D87.2 Intron 1	Traes_5DL_EBE8A2839.2
		Traes_4AL_148DC1816 Exon 7		
		Traes_5BL_8B1E33553.3 Exon 8		
		Traes_4AL_B87BD08F0.2 Exon 4		
		Traes_1DS_49778D85D.2 Exon 5		
		Traes_2BL_1BA046AFF.1 Exon 2		
$(GA)_5$	5	Traes_4AS_8E594F267.1 Exon 5	Traes_2AS_621B8FE92.1 Intron 1	Traes_4AL_D0DECE300.2
				Traes_4AS_8E594F267.1Utr5
				Traes_5AL_FFD2F60A3
$(GA)_7$	4	Traes_4BL_AC29E521A.1 Exon 4	Traes_1DS_92D0315C6.2 Intron 1	Traes_4BL_AC29E521A.1 Utr5
		Traes_1BL_B9305B451.1 Exon 3		
$(GA)_8$	1	Traes_5BL_91FBE67D3.1 Exon 3		
$(CT)_4$	11	Traes_2DL_E566C63F1 Exon 9	Traes_4AL_2FF7E3B18.1 Intron 1	Traes_3AL_61D8E92DB
		Traes_6BS_B7598F2A2 Exon 13	Traes_5AL_60C44B0CA.1 Intron 3	
		Traes_6BS_B7598F2A21.1 Exon 10	Traes_1AL_CEA78C84D.2 Intron 1	
		Traes_7DS_9073D0928 Exon 2	Traes_2BL_FE3AD6919.2 Intron 1	
			Traes_1DS_49778D85D.2 Intron 4	
			Traes_2AS_0607B91CB.2 Intron 1	
$(CT)_5$	1		Traes_4AL_FE0C5AEF4.1 intron 2	
$(CT)_6$	1		Traes_1BS_CEBFF8BEB.1 Intron 1	
$(CT)_9$	1		Traes_1BS_CEBFF8BEB.1 Intron 1	
$(CT)_{12}$	2		Traes_5BL_88D1A0D5E.1 Intron 1	
			Traes_7DS_9073D0928.1 Intron 3	
$(CT)_{21}$	1		Traes_6DS_E66547E66.1 Intron 1	

Table 5. Putative STF interacting proteins identified in a 2174 Y2H library

Clone Code Number	Putative Annotation	Corresponding IWGSC Sequences	Closest proteins in GenBank[a]
YL7819,<br>YL7504	Strubeling-recptor family 6<br>LRR receptor-linked protein kinase	chr7BL contig6729206;<br>chr5BL contig10887838	*EMS53763;*<br>*EMT18352*[d]
YL7507, YL7796	Serine/threonine-protein kinase HT1	chr5BL contig10924391	EMS55472
YL7513, YL7744<br>YL7745, YL7756<br>YL7805	Triticain alpha	chr2AL contig6390523	BAF02546
YL7726, YL7766	40S ribosomal protein S8	chr2BS contig5223496	*EMS65111*
YL7729, YL7734,<br>YL7760, YL7779,<br>YL7797, YL7813,<br>YL7816	Aspartic proteinase nepenthesin-2	chr6AL contig5826979	EMS52672
YL7731,YL7762	Subtilisin-like protease	chr2BL contig8084582	EMS67510
YL7733,YL7741	Papain-like cysteine proteinase (relation to Triticain cysteine)	chr5DL contig4603657	CAQ00103[b]
YL7736, YL7780	Sucrose:fructan 6-fructosyltransferase	chr4AL contig7168216	ACH73192
YL7737, YL7749,<br>YL7757, YL7791,<br>YL7815, YL7817	Stress responsive protein	chr4AS contig6004838	*AFN10736*
YL7744, YL7805	Triticain beta 2	chr2DL contig9908398	ACI00280**[b]**
YL7745, YL7756	Triticain gamma	chr5DL contig4519805	BAF02548
YL7751, YL7773	LEC14B protein	chr1AS contig3301983	EMS68020
YL7753, YL7765,<br>YL7807	CBS domain-containing	chr4DS contig2307992	*ABF98755*[c]
YL7755, YL7763,<br>YL7785, YL7800	Carbon catabolite-derepressing protein kinase	chr4DL contig14454623	**EMT14983**[d]
YL7759,YL7808	Blue copper protein-like	chr6DL contig3243201	*XP_003575544*[e]
YL7505	Glutelin type-A 2 (relation to Triticain cysteine)	chr1DS contig1913584	**EMT13510**[d]
YL7506	Ultraviolet-B receptor UVR8	chr7AS contig4191962	*XP_003574510*[e]
YL7509	Elongation factor 2	chr5BL contig10880690	**EMS67172**
YL7510	Osmotin-like protein	chr3B contig10518626	*XP_003564582*[e]
YL7511	1-aminocyclopropane-1-carboxylate oxidase-1	chr4DL contig14223512	*EMT31795*[d]
YL7728	Glyceraldehyde-3-phosphate dehydrogenase B, chloroplastic	chr4DL contig14365832	**EMS49604**
YL7730	Alcohol dehydrogenase class-3	chr6DL contig3279849	**EMS58027**
YL7738	Eukaryotic translation initiation factor 3	chr6BS contig2936822	*XP_006653944*[c]
YL7740	Cyclophilin A-2	chr6AS contig4374443	AAK49427
YL7742	Cytochrome P450	chr5BS contig2297308	AAN85862
YL7748	Manganese-dependent ADP-ribose/CDP-alcohol diphosphatase	chr7DS contig3868517	EMT26454[d]
YL7752	3-oxoacyl-[acyl-carrier-protein] synthase I, chloroplastic	chr7AS contig4247590	*EMS61772*
YL7754	Senescence-associated family	chr2BL contig8009126	*XP_006385590*[f]
YL7761	Glycosyl hydrolase family protein	chr2BS contig5244778	EMS60749[g]
YL7764	Guanine nucleotide-binding protein subunit beta-like protein A-like	chr3AL contig4310442	*XP_004969582*[h]
YL7768	RING-H2 finger protein ATL28	chr2AL contig6307097	*EMS54905*
YL7769	23 kDa jasmonate-induced protein-like	chr2DS contig472758	*XP_010686329*[i]

YL7770	Thioredoxin superfamily	chr7BS contig3106508	*XP_013460328*[j]
YL7772	Phosphoinositide phospholipase C 6-like	chr5AL contig2769576	XP_003579061[e]
YL7775	60S ribosomal protein L6	chr4AL contig7103384	EMS62493
YL7776	Carboxyl-terminal peptidase precursor	chr5AL contig2734504	*NP_001152099*[g]
YL7777	Rhamnogalacturonate lyase	chr7AS contig4246819	*EMT23951*[d]
YL7778	Guanine nucleotide-binding protein beta subunit-like protein	chr3AL contig4310442	*XP_003569582*[e]
YL7782	Probable protein phosphatase 2C 9	chr3B contig10438686	*EMS67545*
YL7783	Ubiquitin receptor RAD23d	chr5AL contig2777049	XP_003578094[e]
YL7784	DPP6 N-terminal domain-like protein	chr6BS contig3044976	*EOY36187*[k]
YL7786	Centromere protein V-like	chr5BL contig10846740	EMS55525
YL7788	Chlorophyll a/b binding protein (Lolium perenne)	chr1DL contig2250675	*XP_015064801*[l]
YL7790	Dihydroflavonol 4-reductase	chr1DL contig2290990	**EMS58829**
YL7792	GDSL esterase/lipase	chr7DS contig3962837	*EMT15617*[d]
YL7793	NADH ubiquinone oxidoreductase B22-like subunit	chr7DL contig3329305	XP_003563562[e]
YL7794	Cytochrome b6-f complex iron-sulfur subunit	chr2BS contig5208086	*XP_003562932*[e]
YL7795	Auxilin-like protein 1-like	chr3AL contig4319355	*XP_009392411*[k]
YL7798	Phosphomethylpyrimidine synthase	chr4AL contig7166016	**EMS46732**
YL7799	Carbon catabolite-derepressing protein kinase	chr4AL contig7166016	**EMS46732**
YL7801	Peroxidase 66 precursor	chr4DL contig14392605	*XP_004985934*[h]
YL7802	Quinolinate synthase, chloroplastic	chr5DS contig2743262	*XP_003578704*[e]
YL7803	Polypyrimidine tract-binding protein-1-like protein	chr4AS contig6012438	EMS67773
YL7804	Actin, partial	chr1BS contig3414182	**AHE76166**
YL7806	Beta-D-xylosidase	chr6DL contig3284745	BAD06320
YL7809	D-tagatose-1,6-bisphosphate aldolase subunit kbaZ	chr4DL contig14447877	*NP_001078014*[m]
YL7810	Polygalacturonase-1 non-catalytic beta subunit precursor	chr4DS contig180062	*ACG28762*[g]
YL7812	GDSL esterase/lipase	chr4DS contig2278293	**EMT10908.1**[d]
YL7814	Glycosyl hydrolase family 10 protein	chr4DL contig14465167	*XP_003561212.2*[e]
YL7818	Prefoldin_subunit_beta	chr2AL contig1360312	*XP_006647310.1*[c]

[a] More than 95% identity to protein sequences deposited in GenBank, 100% identity to proteins with bold ID or less than 95% identity to proteins with italic ID.

[b] Protein in barley, [c] protein in rice, [d] protein in tausch's goatgrass, [e] protein in still brome, [f] protein in black cottonwood, [g] protein in maize, [h] protein in foxtail millet, [i] protein in beet, [j] protein in medicago, [k] protein in banana, [l] protein in tomato, [m] protein in Arabidopsis, and all others in wheat.

Table S6. Primers for protein-protein and protein-DNA interactions

Primer name	Primer sequence (5'-3') [c]	Vector	Products
STF-BiFC-F1[a]	GGGGACAAGTTTGTACAAAAAAGCAGGCTTCATGTGGATGGT GGGTTAC	pEG101 pEG202-YC	1-242 a.a.
STF-BiFC-R[a]	GGGGACCACTTTGTACAAGAAAGCTGGGTCCCACGTGGCATTCCTTT		
STF-BiFC-F[a]	GGGGACAAGTTTGTACAAAAAAGCAGGCTTCATGGAATCAGC AGCTGCT	pEG101 pEG202-YC	160-358 a.a.
STF-BiFC-R1[a]	GGGGACCACTTTGTACAAGAAAGCTGGGTCGTTTTTCAAGGGAAGAAAC		
SRP-BiFC-F1[a]	GGGGACAAGTTTGTACAAAAAAGCAGGCTTCATGGATGAGGAAGGCTACCC	pEG101 pEG201-YN	244-347 a.a.
SRP-BiFC-R1[a]	GGGGACCACTTTGTACAAGAAAGCTGGGTCCCAGGGGAGGATCTTCCA		
STK-HT-BiFC-F1[a]	GGGGACAAGTTTGTACAAAAAAGCAGGCTTCATGGACAGGAAGAAGTCGGT G	pEG101 pEG201-YN	257-407 a.a.
STK-HT-BiFC-R1[a]	GGGGACCACTTTGTACAAGAAAGCTGGGTCCTTGGAGAAGCCGAAGCAG		
CCDP-BiFC-F1[a]	GGGGACAAGTTTGTACAAAAAAGCAGGCTTCATGGGGGCTGACTATCTACAA TC	pEG101 pEG201-YN	341-509 a.a.
CCDP-BiFC-R1[a]	GGGGACCACTTTGTACAAGAAAGCTGGGTCTAGAACCCTAAGGTTGGTAAG		
SUB-BiFC-F1[a]	GGGGACAAGTTTGTACAAAAAAGCAGGCTTCATGGGGACTCCTGAGAGGGC	pEG101 pEG201-YN	312-461 a.a.
SUB-BiFC-R1[a]	GGGGACCACTTTGTACAAGAAAGCTGGGTCAGCTCGAGCGGTGCCTAG		
6SFT-BiFC-F1[a]	GGGGACAAGTTTGTACAAAAAAGCAGGCTTCATGGACGTGACGAAGCGAGT GATT	pEG101 pEG201-YN	523-616 a.a.
6SFT-BiFC-R1[a]	GGGGACCACTTTGTACAAGAAAGCTGGGTCTTGAACATACGAGTGATC		
STF-GA-EcoRI F1[b]	ATCCG**GAATTC**ATGTGGATGGTGGGT	pMAL-C2	1-90 a.a.
STF-GA1-BamHI-R3[b]	AGGAGA**GGATCC**CTATGCTGCAGATGGATT		
STF-GA-EcoRI F1[b]	ATCCG**GAATTC**ATGTGGATGGTGGGT	pMAL-C2	1-262 a.a.
STF-GA1-BamHI-R1[b]	AGGAGA**GGATCC**CTATGTGGTTGTGGTGGA		
STF-GA-EcoRI F2[b]	ATCCG**GAATTC**ATGGTAACAACTAGAC	pMAL-C2	263-358 a.a.
STF-GA1-BamHI-R2[b]	AGGAGA**GGATCC**TCACAAGGGAAGAAACTC		
STF-EcoRI-F1[b]	CG**GAATTC**ATGTGGATGGTGGGTTACAATGAAG	pMAL-C2	1-358 a.a.
STF -BamHI-R1[b]	CG**GGATCC**TCAGTTTTTCAAGGGAAGAAACTCAATAAACTG		

[a] Primers for protein-protein interactions
[b] Primers for protein-DNA interactions
[c] Boldfaced sequences indicate the restriction enzyme sites for cloning

Table S7. Primers used for EMSA probes and gene expression

Primer name	Primer sequence (5'-3')	Products
SWEET13-BS-AF2	CCTAATCTCTCCCTTCTCTCCAA	210bp
SWEET13-BS-AR2	GATATATAACAGTGAGATGGATGTG	
SWEET13-BS-BF1	TTGCAGATACCAATGCAGTAATGCAA	227bp
SWEET13-BS-BR1	GAGGGATATGAGATGGATGCAAC	
BEL-BS-AF1	GCTTGCGTGTGTGTGCATC	196bp
BEL-BS-AR1	GCTGGGGAGCAGTTGAGC	
BEL-BS-BF1	CTGGTGGCTTGCGTGCA	131bp
BEL-BS-BR1	AAAATGGAGATGCCCACTTGGT	
6SFT-BS-AF1	ACTCCACGCACTAACTTTTTTAATAAGG	189bp
6SFT-BS-AR1	TCCAATCTACGCCACTGTGAAGTG	
6SFT-BS-DF1	AGTACTCCACGCACTAACTAATAAA	179bp
6SFT-BS-DR1	AATCTACGCCACTGTGGAGTC	
ACC-BS-BF1	TTCTTCCAGGTGAATACACAGCAC	185bp
ACC-BS-BR1	CCTTGGTCATCTTCTCCACCTT	
ACC-BS-DF1	TTCTTCCAGGTGAATACACCACAGT	132bp
ACC-BS-DR1	GAGATGCCGTGGTCCAGA	
6SFT-BS-F2	GAGCTTTCTAGAAAATGGTGGAGAC	196bp
6SFT-BS-R1	CCAATATTCTTTCATGACCGCATC	
ABA-BS-AF1	GGCCTTCTCTTAGTTAATTGCACAGA	247bp
ABA-BS-AR1	CTCCGTCAACCCTTGACGA	
KN-BS-ABDF1	TACTGCAGTATTGCCCTCCAGC	192bp
KN-BS-ABDR1	AGACGGATTGAGCTTGAGGTGAAG	
PSR-BS-DF1	CCATTCCAGATTGCATAAGTGCA	230bp
PSR-BS-DR1	ACTGGATCACCCTCTTTTAGTTCG	
TaKN-A1-F1*	GGAGGGTGGAGACGCAACTCAACT	548bp
TaKN-A1-R1*	CACCGACCAAGGTCACCAGT	
Ta6SFT-A1-F1*	AAGGACGTGACGAAGCGA	309bp
Ta6SFT-A1-R1*	CTTCTTTGATCCAATGTAGCTTCATTG	
STF-Exp-F8*	GCAGCAGCAAGCATCAGTAG	134bp
STF-Exp-R8*	ACAATTCTTCCAGTGCTCTTAACTG	
Actin-F2*	GGAACTGGCATGGTCAAGGCTG	107bp
Actin-R2*	CCCATCCCCACCATCACACC	
TaSWEET13-A1-F1*	GACAAATACGTCGCGATGGC	182bp
TaSWEET13-A1-R1*	GCCGGAACGGCGGTGACT	
TaPSR-D1-F1*	GAACGAGCGAAACTCCGAGGTT	229bp
TaPSR-D1-R1*	GTGTATATATTGGGCTGTACCCATCAACA	

* Primers for gene expression

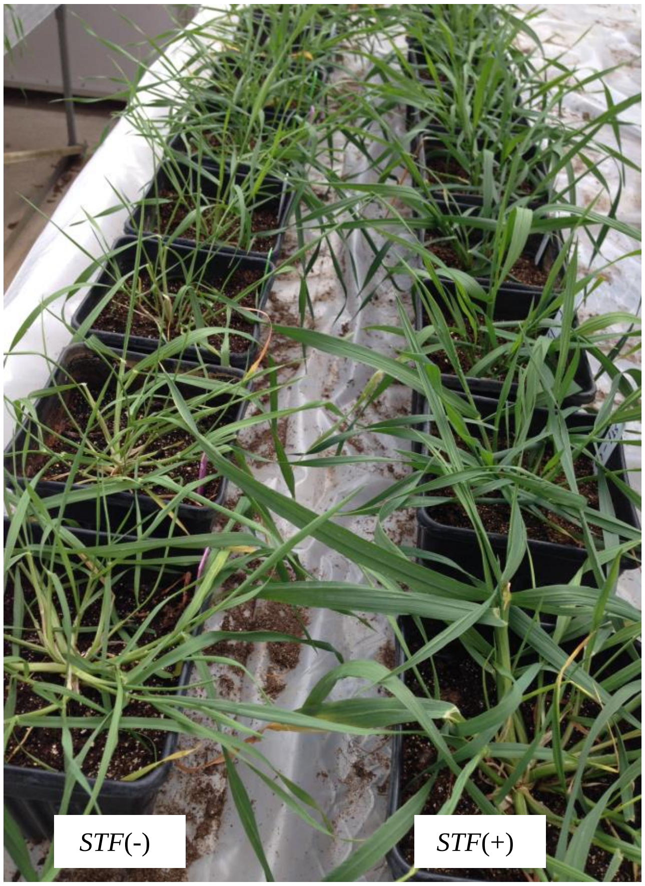

Figure S1 Transgenic T_0 wheat plants expressing *STF*. Images of negative T_0 plants *STF*(-) on the *Left*, and the transgenic plants *STF*(+) on the *Right*. These plants were grown in a greenhouse at constant temperature 20-25°C and with a long day (LD, 16/8 hours light/dark).

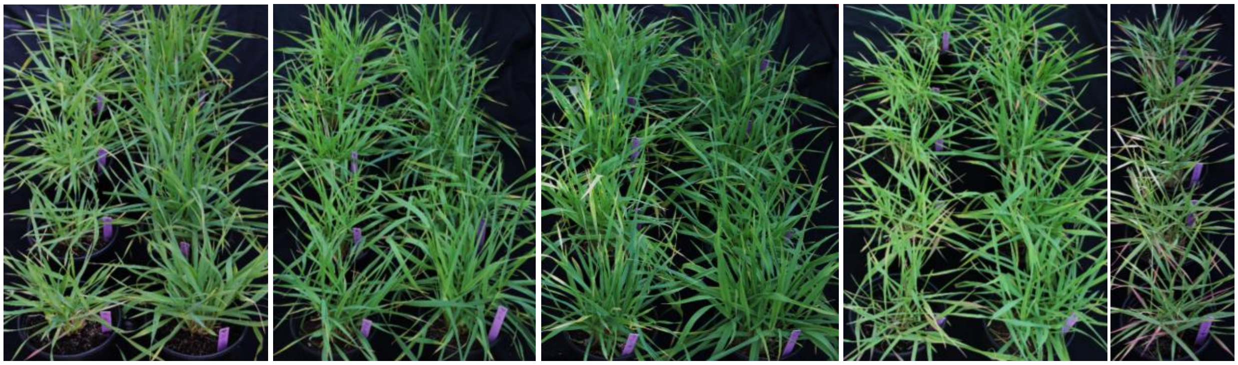

N(-) P(+) STF24
N(-) P(+) STF31
N(-) P(+) STF32
N(-) P(+) STF44
Wild type

Figure S2. Images of representative transgenic T_1 plants. Postive transgenic plants expressing *STF* on the *Right* {P(+)} and its negative non-transgenic sibling on the *Left* {N(-)}. These T_1 plants derived from four positive T_0 plants, STF24, STF31, STF32, and STF44. The positive plants showed wider and more grene leaves than the negative plants and the wild type 2174.

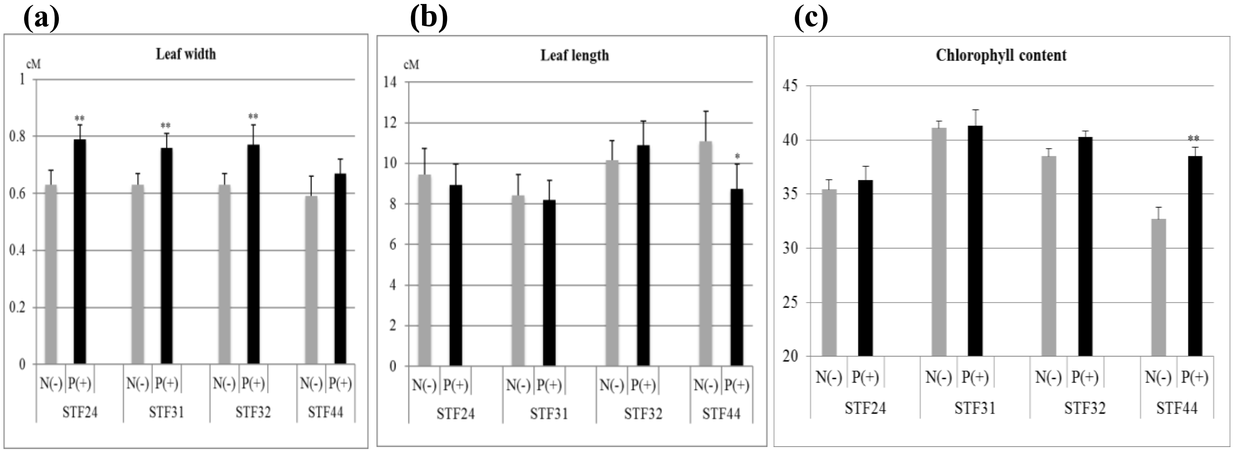

Figure S3 Comparison of phenotypes between the transgenic plants and non-transgenic plants in four T_1 populations. Shown are means ± SD for n = 6-20 plants for transgenic or nontransgnic siblings dervied from each transgenic event. Three largest leaves of each plant were masured for leaf width, leaf length and chlorophyll content. Plants were grown on April 8, 2015, leaf width and length were measured at the adult stage, on June 9 and chlorophyll was measured on July 6, 2015. (a) Leaf width. (b) Leaf length. (c) Chlorophyll content. *$P < 0.05$ and **$P < 0.001$, Student's t-test.

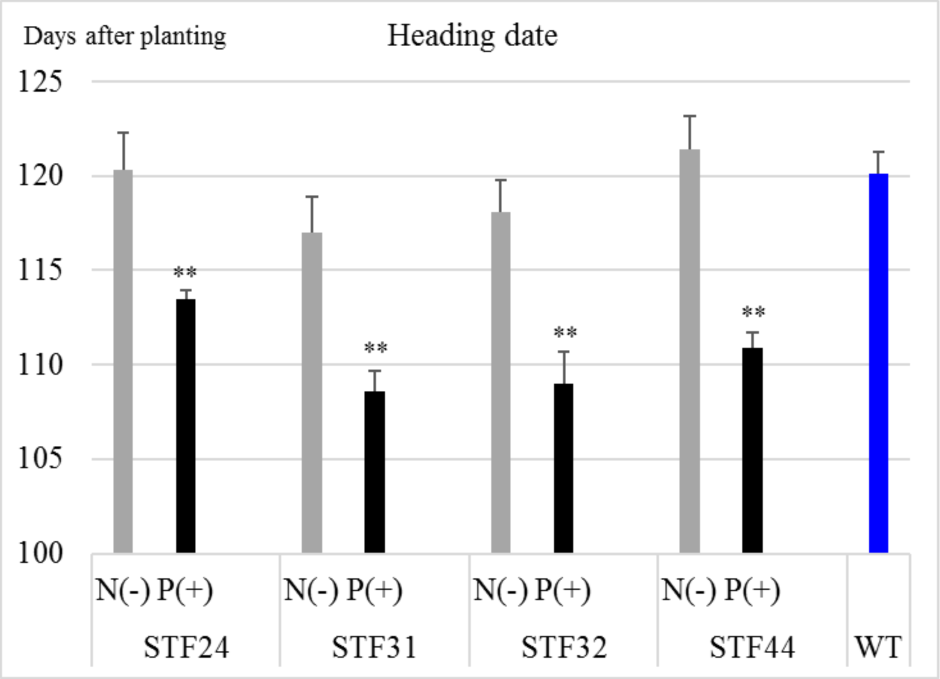

Figure S4. Comparison of heading date between the transgenic plants, non-transgenic plants, and the wild type. The T_2 populations were generated from three positive transgenic T_1 plants and one negative non-transgenic plants for each of transgenic events. These T_2 populations were continuously grown in a greenhouse at constant temperature 20-25°C and with a long day (LD, 16/8 hours light/dark) and without vernalization. Plants were grown on October 30, 2015. Shown are means ± SD for n = 8-16 plants for transgenic or nontransgnic siblings dervied from each transgenic event. **$P < 0.01$, Student's *t*-test.

(a) (b) (c) (d)

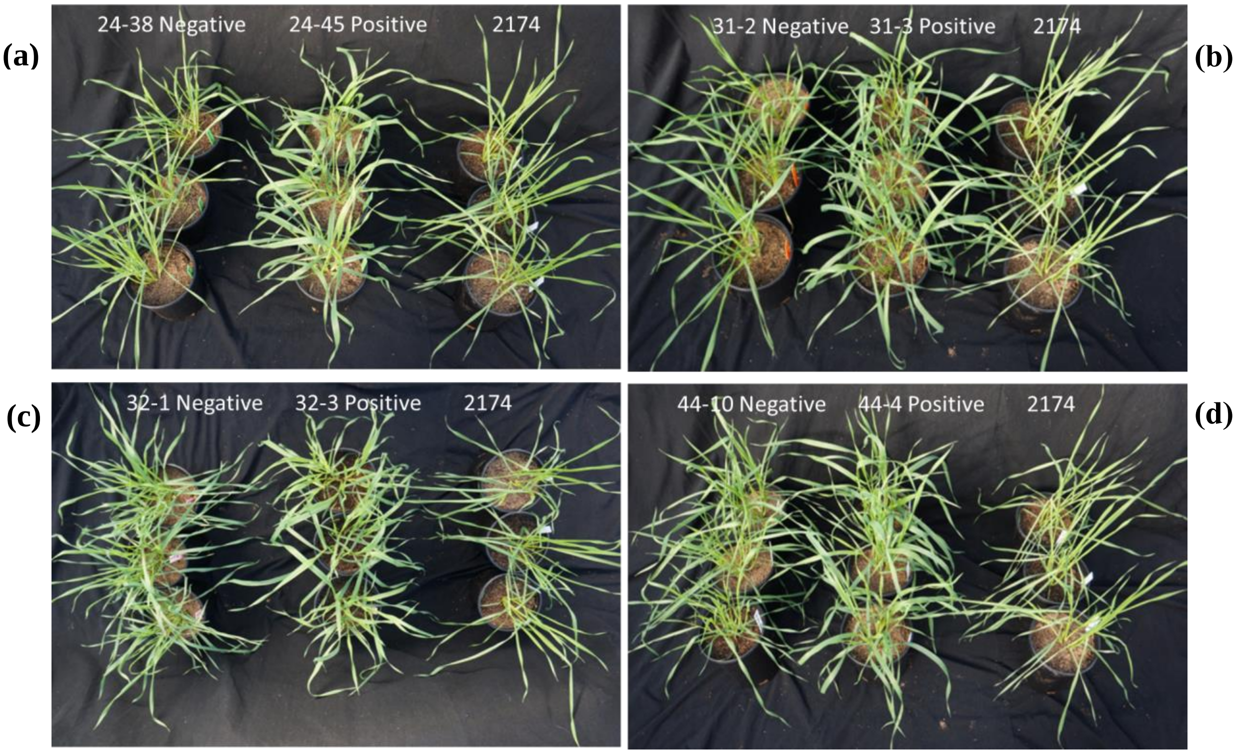

Figure S5. Images of representative transgenic T_2 plants. Three positive T_1 plants derived from each of transgenic events were used to generate T_2 populations, and non-transgenic plants and the wild type were used as controls. The T_2 lines included three positive T_2 lines 31-33, 31-38, 31-45 and one non-transgenic line 24-38 for STF24; three positive T_2 lines 24-34, 24-45, 24-47 and one non-transgenic line 31-25 for STF32; three positive T_2 lines 32-3, 32-11, 32-14 and one non-transgenic line 32-1 for STF32; three positive T_2 lines 44-1, 44-2, 44-4 and one non-transgenic line 44-10 for STF44. These T_2 plants derived from four positive T_1 plants of STF24 (a), STF31 (b), STF32 (c), and STF44 (d). Postive transgenic plants expressing *STF* on the *Middle*, negative non-transgenic sibling on the *Left*, and the wild type on the *Right*.

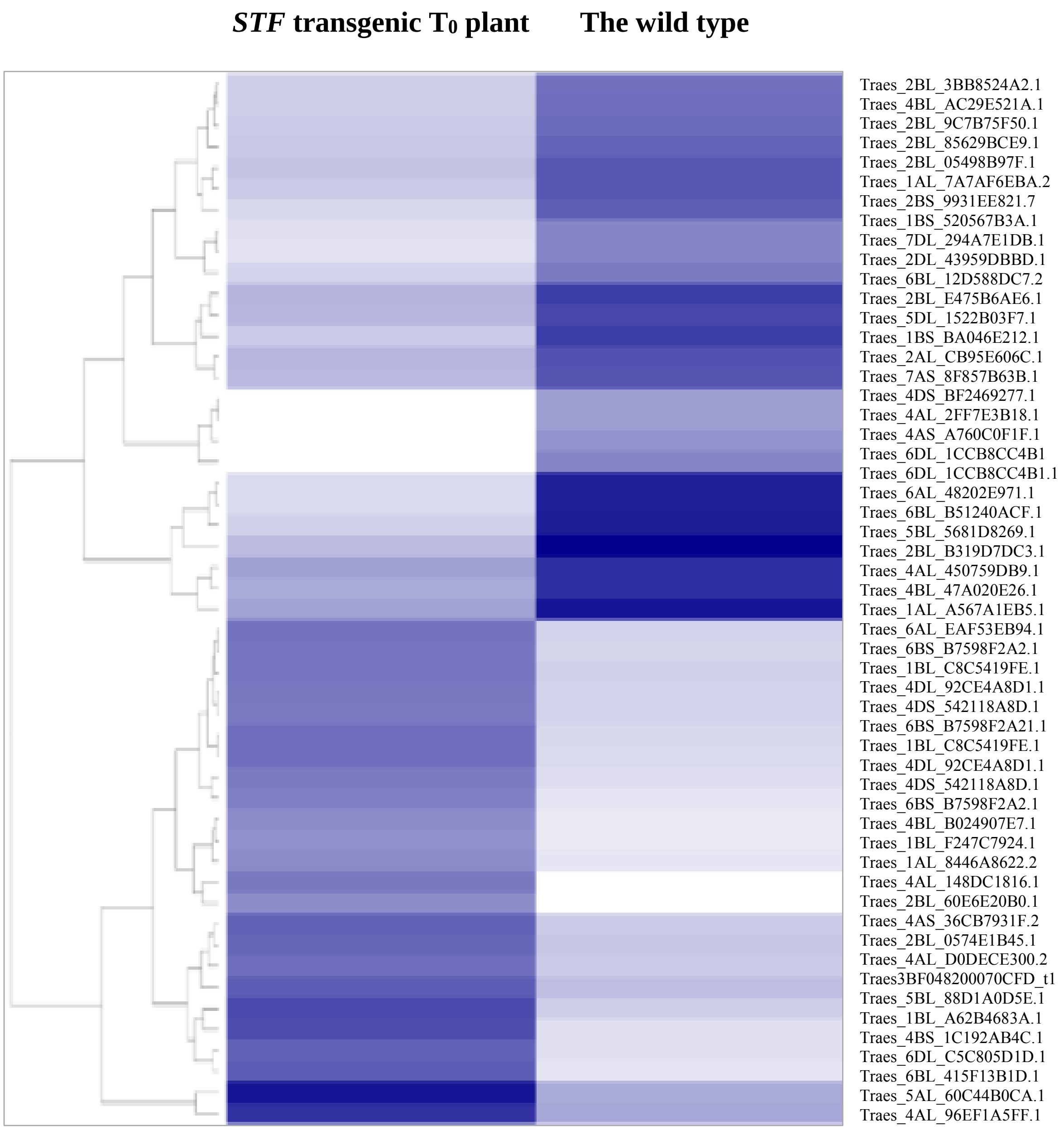

Figure S6 Heatmap of Top 100 Genes/Transcripts by P-value. A paired end read 2x100 bp sequencing runs of RNA libraries were performed using the Illumina HiSeq 2000 instrument. Reads were demultiplexed by barcode and output into FASTQ format for subsequent analysis. Raw sequence data was generated using Illumina BCL2FASTQ. Reads were aligned to *T. aestivum* cDNA sequences using Bowtie2. A total of 118,505,212 reads was obtained for the *STF* sample and 46,486,361 reads for the control. Reads mapping to cDNA sequences were counted, and unreplicated differential expression analysis was performed using DESeq. One hundred ninety-eight cDNAs were identified with a Padj value of less than 0.01.

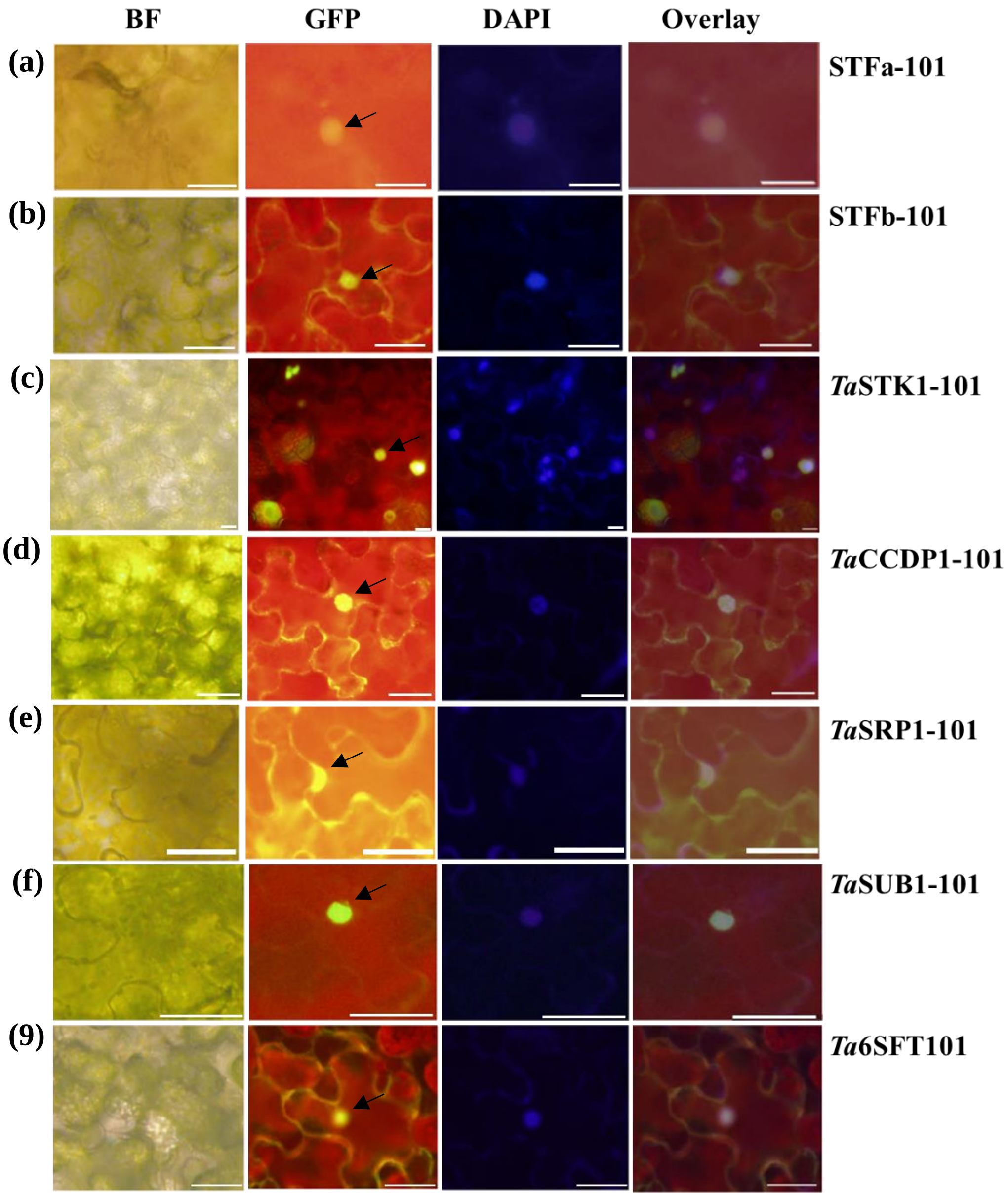

Figure S7 Subcellular localization of STF and its interacting proteins. STFa, and STFb, and each of wheat proteins identified from the Y2H library were expressed in pEG101 vector and then infected into *N. benthamiana* leaves (five weeks old). Leaf discs were imaged three days after infiltration under a fluorescent microscope (Olympus BX51). An black arrow points to stained nucleus and a white arrow on the *Ta*STK1 image point to cytoplasm. (a) STFa. (b) STFb. (c) *Ta*STK1. (d) *Ta*CCDP1. (e) *Ta*SRP1. (f) *Ta*SUB1. (g) *Ta*6SFT1. Images were taken with a bright filter (BF), a GFP filter, and with an ultraviolet filter (DAPI). The overlay images align the locations of YFP with the DAPI-stained nucleus. The scale bar in all images represents 50 μm.

>TaSWEET13-D1

TTGCAGATACCAATGCAGTAATGCAATCTTCCTGCCTATATAAAGCACCCCGTTAGCAACCTTACAATTCAGGCGAAGAGGTTCAACGAACACAGAG
AGCTCCTCTCCAAACCTCTCCCTTCTCCCCGCCAGGAGTCTAGGGTTAGAGAGAGAGAGAGAGAGTGAGACAAGAGCAAAAATAATCTCTCGCCCCA
CTGTTATACCAAGGTTGCATCCATCTCATATCCCTC

>TaSWEET13-A1

CCTAATCTCTCCCTTCTCTCCAACAGGAGTCTGGGGTTAGAGAGAGAGAGAGAGAGAGAGAGAGAGAAAGAGAGAGAGAGAGAGAGAGAGAGAGAGAGA
GAGAGAGAGAGAGAGAGAGAGAGAGAGAGAGAGAGAGAGAGAGAGAGAGAAGTGCAAAAACAATCTCTCGCCCCACTGTTATACCAAGTTCACATCCAT
CTCACTGTTATAT

>TaPSR-D1

ATCCATTCCAGATTGCATAAGTGCAGCGTTTCTGATCAGCCAAGCAAGAGACAAGGGTTGTTCACGTGATCATGGACCCATGACACACCATTATAGC
AACCTTCCAACCTAAAAATAGACCATCAAATCCTTTTCACTCACTGTCTGCCACTCCCCAGCCTCCCTGTATTTAAACCCATGCATTAGCCGTGAGA
GAAACTAAATCGAACTAAAAGAGGGTGATCCAGTGAGCACAAACATGTCTGCCTCTGTCATG

>TaABA-A1

GGCCTTCTCTTAGTTAATTGCACAGAAGCCAGAGAAGAGAGAGAGAGAAGGTGGGAGAGAGAGAGAGAGAGAGAGAGAGAGAGAGAGAGAGAGAGAG
AGAGAGAGAGAGAGAGAGAGAGAGAGAGAGAGAGAGAGAGAGAGAGAGAGAGAGAGAGAGAGAGAGAGAGAGAGAGAGAGAGAGAGAGAGAGAGAGAGA
GGAAGAGCATGGAGCCCCACATGGAGAGCGCGCTTCGTCAAGGGTTGACGGAG

>TaBEL-A1

GCTTGCGTGTGTGTGCATCAGAGAGAAAGAGAGAGAGAGAGAGGCCTGCCGCCCAAGATCTCTCTTGATCAGTGCCGAGATCATCAATCAGGGGGGC
CTGCCATCCAAGTGGGCATCTCCATTTTTCTTCCTCCTCGAGCTGGTTTCCGAGCCTGATAAAACCACCCTCCCCGGCCTCGCTCAACTGCTCCCCA
GC

>TaKN-B1

TACTGCAGTATTGCCCTCCAGCTTTTTCCTCTCGCTCCCCATCTCCTTCCCTCTAATGATAGCTTAGAGAGAGAAGGAGAAAAGGCTATTCCGCTCA
AGGCAAGGCAGGCAGGTACCCTTCTCCTTCCCCAAACCCAAACCCTTTTTCTCTTTCTCTCCGCCCAAGAACTTCACCTCAAGCTCAATCCGTCT

>TaACC-B1

TTCTTCCAGGTGAATACACAGCACTAACATGGCATAATCTGACAGGATAAATCTCCTTATCGATCTATCCTTAATGCTCATCTGCTCTCTCTCTCTC
TCTCTCTCTCTTTTTCGGTGGTGCGTGCAGGTCCTGGACCACGGCATCTCGACGGAGCTGATGGACAAGGTGGAGAAGATGACCAAGG

>TaACC-D1

TTCTTCCAGGTGAATACACCACAGTAGTAATATGGCATAATCTGACAAGATGAATCTCCTCATCGACCCATCCTTAATGTTGATTTGCTCTCTCTCT
GTTTTCGGCGGTGCGTGCAGGTTCTGGACCACGGCATCTC

>Ta6SFT-A1

ACTCCACGCACTAACTTTTTTAATAAGGTCTTCTGTGTGGGGAAAAAGACGGGATCTCTCTCTCTAGACATAATCAAAAGGGATTGTTTAACTCACA
CCAAATTTGGAGGAAAACTTTGACAGCAAAACAGGGGAAAACAGAGTTTCCATAGCGGGTTGGGGGTTCACTTCACAGTGGCGTAGATTGGA

Figure S8. The complete sequences of the DNA probes used for interactions with STF
